# Identification of Novel Low-Dose Bisphenol A Targets in Human Foreskin Fibroblast Cells Derived from Hypospadias Patients

**DOI:** 10.1371/journal.pone.0036711

**Published:** 2012-05-04

**Authors:** Xian-Yang Qin, Yoshiyuki Kojima, Kentaro Mizuno, Katsuhiko Ueoka, Koji Muroya, Mami Miyado, Hiroko Zaha, Hiromi Akanuma, Qin Zeng, Tomokazu Fukuda, Jun Yoshinaga, Junzo Yonemoto, Kenjiro Kohri, Yutaro Hayashi, Maki Fukami, Tsutomu Ogata, Hideko Sone

**Affiliations:** 1 Health Risk Research Section, Research Center for Environmental Risk, National Institute for Environmental Studies, Tsukuba, Ibaraki, Japan; 2 Department of Environmental Studies, Graduate School of Frontier Science, The University of Tokyo, Kashiwa, Chiba, Japan; 3 Department of Nephro-Urology, Nagoya City University Graduate School of Medical Sciences, Nagoya, Aichi, Japan; 4 Department of Surgical Subspecialties, National Research Center for Child Health and Development, Tokyo, Japan; 5 Division of Endocrinology and Metabolism, Kanagawa Children's Medical Center, Kanagawa, Yokohama, Japan; 6 Department of Endocrinology and Metabolism, National Research Institute for Child Health and Development, Tokyo, Japan; 7 Department of Animal Production Science, Graduate School of Agricultural Science, Tohoku University, Sendai, Miyagi, Japan; 8 Department of Pediatrics, University Hospital, Hamamatsu University School of Medicine, Hamamatsu, Shizuoka, Japan; Baylor College of Medicine, United States of America

## Abstract

**Background/Purpose:**

The effect of low-dose bisphenol A (BPA) exposure on human reproductive health is still controversial. To better understand the molecular basis of the effect of BPA on human reproductive health, a genome-wide screen was performed using human foreskin fibroblast cells (hFFCs) derived from child hypospadias (HS) patients to identify novel targets of low-dose BPA exposure.

**Methodology/Principal Findings:**

Gene expression profiles of hFFCs were measured after exposure to 10 nM BPA, 0.01 nM 17β-estradiol (E2) or 1 nM 2,3,7,8-tetrachlorodibenzo-p-dioxin (TCDD) for 24 h. Differentially expressed genes were identified using an unpaired Student's t test with *P* value cut off at 0.05 and fold change of more than 1.2. These genes were selected for network generation and pathway analysis using Ingenuity Pathways Analysis, Pathway Express and KegArray. Seventy-one genes (42 downregulated and 29 upregulated) were identified as significantly differentially expressed in response to BPA, among which 43 genes were found to be affected exclusively by BPA compared with E2 and TCDD. Of particular interest, real-time PCR analysis revealed that the expression of matrix metallopeptidase 11 (MMP11), a well-known effector of development and normal physiology, was found to be inhibited by BPA (0.47-fold and 0.37-fold at 10 nM and 100 nM, respectively). Furthermore, study of hFFCs derived from HS and cryptorchidism (CO) patients (*n* = 23 and 11, respectively) indicated that MMP11 expression was significantly lower in the HS group than in the CO group (0.25-fold, *P* = 0.0027).

**Conclusions/Significance:**

This present study suggests that an involvement of BPA in the etiology of HS might be associated with the downregulation of MMP11. Further study to elucidate the function of the novel target genes identified in this study during genital tubercle development might increase our knowledge of the effects of low-dose BPA exposure on human reproductive health.

## Introduction

Hypospadias (HS) is one of the most common congenital abnormalities with a global prevalence of approximately 0.2–1% at birth in male infants [1]. The etiology of HS is poorly understood, and might include genetic, hormonal and environmental factors. It has been hypothesized that testicular cancer, cryptorchidism (CO) and some cases of HS and impaired spermatogenesis are symptoms of a single underlying entity that has been named as the testicular dysgenesis syndrome (TDS) [2], [3]. This concept proposes the existence of a common underlying cause for the occurrence of these reproductive and developmental diseases, and suggests that adverse environmental factors, such as environmental endocrine disruptors (EEDs) might exert their etiological effects on a susceptible genetic background.

Bisphenol A (BPA) is one of the world's highest production-volume chemicals, with more than six billion pounds produced worldwide each year [4]. BPA is used extensively in the plastics produced for food and beverage containers, such as baby bottles, plastic containers and the resin lining of cans [4]. Among the known estrogen-like EEDs, BPA has received much attention because it is commonly found in the environment as well as in human tissues and fluids (1–19.4 nM) [4], [5]. BPA has been detected in 92% of urine samples in a US reference population, suggesting people may be continuously exposed to this compound in their daily lives [6]. The US Food and Drug Administration and Environmental Protection Agency concluded in the 1980s that a daily dose of 50 µg/kg/day was safe for humans, which is currently considered as <2.19×10^−7^ M for *in vitro* cell or organ culture studies [7]. However, in recent decades, there has been a heated controversy over the safety of BPA among scientists and risk assessors.

Recently, exposure to BPA at concentrations detected in humans has been reported to affect neurological, cardiovascular and metabolic diseases (such as diabetes), and even cancers [8]–[12]. However, the effect of low-dose BPA exposure on human reproductive health is still controversial [13], [14]. Li *et al.* reported that occupational exposure to BPA has adverse effects on male sexual dysfunction, which is the first evidence that exposure to BPA in the workplace could have an adverse effect on male sexual dysfunction [15]. Jasarevic *et al.* reported that exposure to BPA at low doses can affect sexual behaviors, even with no changes in sexual phenotypes or hormones [16]. Furthermore, Zhang *et al.* reported that low-dose BPA exposure could directly disrupt steroidogenesis in human cells [17]. It seems that exposure to BPA might affect human reproductive health by complicated mechanisms that encompass more than just estrogen receptor (ER) mediated pathways.

In this study, to better understand the molecular basis of the effects of BPA on human reproductive health, a genome-wide screen was performed using human foreskin fibroblast cells (hFFCs) derived from child HS patients to identify novel targets of low-dose BPA exposure. Furthermore, the effect of BPA on the global gene expression profile of hFFCs was compared with that of 17β-estradiol (E2) and 2,3,7,8-tetrachlorodibenzo-p-dioxin (TCDD), which are representative agonists of ER and aryl hydrocarbon receptor (AhR) signaling pathways, respectively.

## Materials and Methods

### Samples

hFFCs from child HS (*n* = 23; median age 2.3 yrs) and CO (*n* = 11; median age 2.3 yrs) patients undergoing surgical procedures were obtained from the National Research Institute for Child Health and Development, Japan, during 2007–2009. All subjects were of Japanese origin and written informed consent was obtained from the guardians on the behalf of the children participants involved in this study. This study was approved by the Institutional Ethics Committees of the Nagoya City University Graduate School of Medical Sciences, the National Research Institute for Child Health and Development and the National Institute for Environmental Studies.

### Chemicals

Dimethyl sulfoxide (DMSO) and E2 were obtained from Sigma Chemical Co. (St. Louis, MO, USA). BPA was obtained from Wako Industries (Osaka, Japan) and TCDD was obtained from Cambridge Isotope Laboratories (Cambridge, MA, USA). DMSO was used as the primary solvent for all chemicals, and the DMSO solutions were further diluted in cell culture media for treatments. The final concentrations of DMSO in media did not exceed 0.1% (vol/vol).

### Cell culture

hFFCs were maintained in Dulbecco's Modified Eagle Medium (DMEM)/Ham's F-12 (048-29785, Wako, Osaka, Japan) containing 10% fetal bovine serum (FBS, Mediatech, Herndon, VA, USA) and grown at 37°C in a 5% CO_2_ humidified incubator. For growth under steroid-free conditions, cells were seeded in phenol red-free DMEM/Ham's F-12 (045-30665, Wako) containing 5% charcoal/dextran-treated FBS (Hyclone, Logan, UT, USA). All culture media contained 100 U/ml penicillin/streptomycin and 2 mmol/L _L_-glutamine (Mediatech, Herndon, VA, USA).

### RNA isolation and DNA microarray analysis

Total RNA was isolated from cultured cells after treatment with chemicals for 24 h using an RNeasy Kit (Qiagen, Valencia, CA, USA) in accordance with the manufacturer's instructions. Quantification and quality assessment of the isolated RNA samples were performed and verified using an Agilent Bioanalyzer2100 (Agilent Technologies, Palo Alto, CA, USA) and a NanoDrop spectrophotometer (NanoDrop products, Wilmington, DE, USA) in accordance with the manufacturer's instructions. RNA was amplified into cRNA and labeled according to the Agilent One-Color Microarray-Based Gene Expression Analysis protocol (Agilent Technologies). Samples were then hybridized to G4851A SurePrint G3 Human GE 8×60K array slides (60,000 probes, Agilent Technologies). The slides were processed according to the manufacturer's instructions without any modification. The arrays were scanned using an Agilent Microarray Scanner (G2565BA, Agilent Technologies).

### MIAME

All data are MIAME compliant, and the raw data have been deposited in the Gene Expression Omnibus (www.ncbi.nlm.nih.gov/geo, accession no. GSE35034).

### Array data analysis

The scanned images were analyzed using the standard procedures described in the Agilent Feature Extraction software 9.5.3.1 (Agilent Technologies). Data analysis was performed with GeneSpring GX12.0.2 (Agilent Technologies). Signal intensities for each probe were normalized to the 75th percentile without baseline transformation. Genes that were differentially expressed following chemical treatments were identified by the unpaired Student's t test with P values cut off at 0.05 and fold change of more than 1.2 and were used for the network generation and pathway analysis.

### Network generation and pathway analysis

The Ingenuity Pathways Analysis (IPA) program (Ingenuity Systems, Mountain View, CA, USA; http://www.ingenuity.com) was used to identify networks and canonical pathways of genes differentially expressed in response to BPA, E2 and TCDD. IPA software uses an extensive database of functional interactions that are drawn from peer-reviewed publications and manually maintained [18]. For the IPA analysis, the Agilent SurePrint G3 Human GE 8×60 K Array was used as a reference gene set. The generated biological networks were ranked by score, which is the likelihood of a set of genes being found in the networks owing to random chance, identified by a Fisher's exact test. The generated canonical pathways were ranked by P values, which is calculated using a Fisher's exact test by comparing the number of user-specified genes of interest that participate in a given function or pathway, relative to the total number of occurrences of these genes in all functional/pathway annotations stored in the Ingenuity Pathways Knowledge Base [19]. In addition, genes significantly differentially expressed in response to BPA, E2 and TCDD was analyzed by Pathway Express (http://vortex.cs.wayne.edu/projects.htm) and mapped to Kyoto Encyclopedia of Genes and Genomes (KEGG) pathways by KegArray (http://www.kegg.jp/kegg/download/kegtools.html).

### Quantitative real-time reverse-transcription polymerase chain reaction (RT-PCR)

cDNA was synthesized using a High Capacity RNA-to-cDNA Kit (Applied Biosystems, Foster City, CA, USA) according to the manufacturer's instructions. Real-time PCR was performed using TaqMan® Gene Expression Master Mix (Applied Biosystems) in accordance with the manufacturer's instructions. TaqMan® Gene Expression Assays (Applied Biosystems) used in this study were: Hs02341150_m1 for POMZP3, Hs01094348_m1 for WDR3, Hs00171829_m1 for metallopeptidase 11 (MMP11; see gene names in Table S1), and Hs00266705 g1 for glyceraldehyde-3-phosphate dehydrogenase (GAPDH). The primers (Forward: 5′-TGTTGGGGGATAAGGACAAA-3′; and Reverse: 5′-GCAGGCTGTACAGGAACCAT-3′) and probe (5′-TAAACTCACCTCTGTGGTTGGAACAAT-3′) for NEK10 were designed and synthesized by Hokkaido System Science (Sapporo, Hokkaido, Japan). The amplification reaction was performed in an ABI PRISM 7000 Sequence Detector (Applied Biosystems) under the following cycling conditions: 95°C for 15 min, followed by 40 cycles of 95°C for 15 s and 60°C for 60 s. The gene expression levels were calculated based on the threshold cycle using Sequence Detection System Software (Applied Biosystems). Gene expression was normalized to that of GAPDH and set to 100 for the control DMSO-treated cells.

### Statistical and multivariate analysis

Quantitative data were expressed as the mean ± SEM. A nonparametric test, the Mann-Whitney U test, was applied to test for statistical significance. Values of *P*<0.05 were considered to indicate statistical significance. Unsupervised principal component analysis (PCA) was run in SIMCA-P+ (Version 12.0, Umetrics, Umeå, Sweden) to obtain a general overview of the variance of genes differentially expressed in response to BPA, E2 and TCDD.

## Results

### Gene expression profiles of hFFCs in response to BPA, E2 and TCDD

The gene expression profiles in hFFCs treated with DMSO control or 10 nM BPA, 0.01 nM E2 or 1 nM TCDD were determined by Agilent microarray analysis using three biological replicates. Then, differentially expressed genes in response to BPA, E2 and TCDD compared with DMSO control were identified by the unpaired Student's t test with *P* values cut off at 0.05 and fold change of more than 1.2 using GeneSpring GX software. Seventy-one genes (42 downregulated and 29 upregulated), 814 genes (371 downregulated and 443 upregulated), and 824 genes (344 downregulated and 480 upregulated) were identified to be significantly differentially expressed in response to BPA, E2, and TCDD, respectively. No nuclear receptor was found to be significantly differentially expressed in response to BPA, while estrogen-related receptor-α (ESRRA), retinoic acid receptor-α (RARA) and RAR-related orphan receptor-α (RORA) and RARA were found to be significantly differentially expressed in response to E2 and TCDD, respectively. The summary of differentially expressed genes along with their *P* values and fold changes is provided in Table 1.

**Table 1:** Summary of genes differentially expressed in response to BPA, E2 and TCDD.

	BPA		E2		TCDD	
P-value	1.0-fold	1.2-fold	1.0-fold	1.2-fold	1.0-fold	1.2-fold
0.05	154	71*	1101	814*	1150	824*
0.01	30	17	198	154	208	156
0.001	7	5	16	11	14	9

* Selected as significant differentially expressed genes and used for the network generation and pathway analysis.

### Differences in the response of hFFCs to BPA, E2 and TCDD

**Figure 1:**
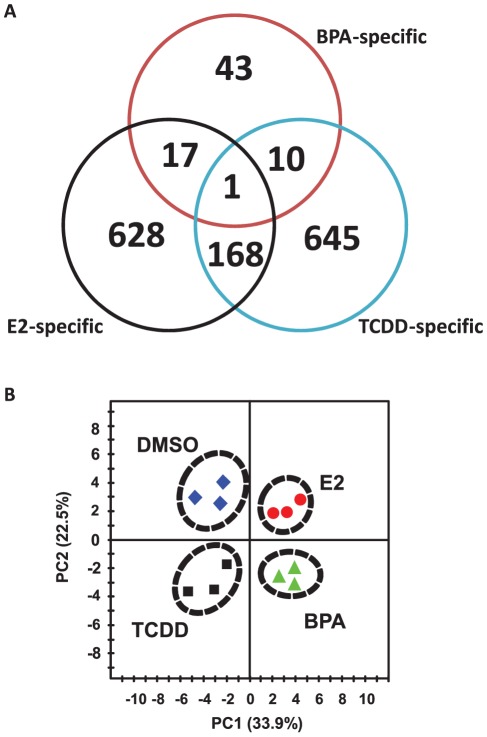
Genetic response of hFFCs to BPA, E2 and TCDD. (***A***) Venn-diagrams showing the number of genes that were considered significantly deregulated among the three treatment groups. (***B***) PCA scoreplot from transcript data of three hFFC cultures treated with DMSO, 10 nM BPA, 0.01 nM E2 and 1 nM TCDD.

Comparison of the gene expression profiles of hFFCs in response to BPA, E2 and TCDD is provided in Figure 1. BPA-specific responses were found in 43 significantly differentially expressed genes, compared with responses to E2 and TCDD (Figure 1A). Seventeen and 10 differentially expressed genes were found to be common in response to BPA with E2 or TCDD, respectively. A full list of these genes is summarized in Table S1.

Furthermore, to compare the expression patterns of hFFCs in response to BPA with that of E2 or TCDD, PCA analysis was performed on the data of significantly differentially expressed genes in response to BPA. PCA is a standard technique of pattern recognition and multivariate data analysis. Of interest, the cells treated with DMSO, BPA, E2 and TCDD were clearly distinguished from each other by the PCA score plots (Figure 1B). According to the first component (PC1), which represents 33.9% of the total variance, a very clear discrimination between cells treated with BPA or E2 and those treated with DMSO or TCDD was observed. However, according to the second component (PC2), which represents 22.5% of the total variance, cells treated with BPA or TCDD were clearly distinguished from those treated with DMSO or E2. It should be noted that differences in the PCA were identified using an unsupervised analysis, without any prior information on the samples. Since all cells were cultured under identical conditions, the observed discriminations demonstrate that the effect of BPA is similar to that of E2 according to PC1 but is similar to that of TCDD according to PC2.

### Network generation and pathway analysis of genes differentially expressed in response to BPA, E2 and TCDD

**Figure 2:**
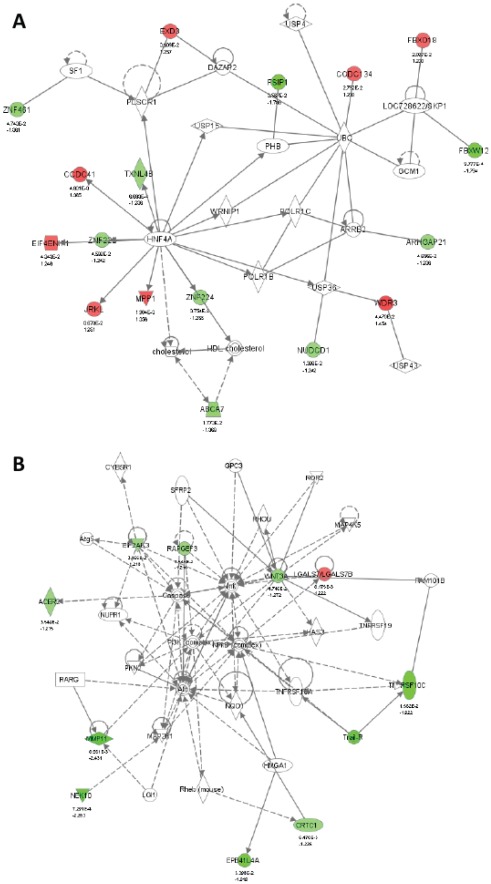
Network associated genes differentially expressed in response to BPA. (***A***) “Endocrine System Disorders, Gastrointestinal Disease, Genetic Disorder” network and (***B***) “Cell Death, Cellular Growth and Proliferation, Cancer” network. The images were created using the IPA platform by overlaying the differentially expressed genes in response to BPA detected by Agilent microarray analysis onto a global molecular network from the Ingenuity knowledgebase. Red indicates upregulated genes, green indicates downregulated genes, and white indicates genes that were not annotated in this array but that form part of this network. The bottom numbers indicate the fold changes induced by BPA, and the top numbers are the *P*-values between the DMSO control group and the BPA treated group. Direct relationships are exhibited with solid arrows and indirect relationships with dashed arrows.

To investigate possible biological interactions of differently regulated genes, datasets derived from microarray analysis representing genes with altered expression profiles were imported into the IPA platform. Network analysis of the biological functions of the top five IPA-generated networks is summarized in Table 2 and is shown in Figure 2 and Figure S1,S2,S3.The two most highly populated biological networks entitled “Endocrine System Disorders, Gastrointestinal Disease, Genetic Disorder” (Score = 41) and “Cell Death, Cellular Growth and Proliferation, Cancer” (Score = 21) were identified with genes differentially expressed in response to BPA (Figure 2). The networks consisted of genes that encoded enzymes (ACER2, PLSCR1, POLR1C, TXNL4B and UBC), peptidases (MMP11, UCHL5, USP4, USP36 and USP43), proteins that regulate transcription (ABCA7, CRTC1, HNF4A, LOC728622/SKP1, PHB, SF1 and SLC25A6) and translation (EIF4ENIF1 and TNFRSF10C), and others (ARHGAP21, ARRB2, CCDC41, CCDC134, EIF2AK3, EPB41L4A, DAZAP2, EPB41L3, EXD3, FBXO18, FBXW12, FSIP1, JRKL, LGALS7/LGALS7B, NEK10, NUDCD1, RAPGEF3, SERPINA1, WDR3, WNT3A, ZNF222, ZNF224 and ZNF461). The most highly populated biological networks were identified with genes differentially expressed in response to E2 and TCDD and were entitled “Cellular Growth and Proliferation, Skeletal and Muscular System Development and Function, Cell Cycle” (Score = 41) and “Post-Translational Modification, Genetic Disorder, Hematological Disease” (Score = 49), respectively. Furthermore, top canonical pathways associated with genes significantly differentially expressed in response to BPA, E2 and TCDD were summarized in Table 3. The pathway most affected by BPA is “RAN Signaling” with only borderline significance (*P* = 0.0531). The pathways most affected by E2 and TCDD are “Cell Cycle: G1/S Checkpoint Regulation” and “Cell Cycle Control of Chromosomal Replication”, respectively (*P* = 1.01×10^−3^ and 1.20×10^−9^, respectively).

**Table 2:** Top five associated network functions of genes differentially expressed in response to BPA, E2 and TCDD generated by IPA.

Chemical	Top Functions	Score
BPA	Endocrine System Disorders, Gastrointestinal Disease, Genetic Disorder	41
	Cell Death, Cellular Growth and Proliferation, Cancer	21
	Cellular Growth and Proliferation, Hematological System Development and Function, Cellular Development	18
	Cellular Assembly and Organization, Cellular Function and Maintenance, Cell Cycle	13
	Dermatological Diseases and Conditions, Inflammatory Disease	3
E2	Cellular Growth and Proliferation, Skeletal and Muscular System Development and Function, Cell Cycle	41
	DNA Replication, Recombination, and Repair, Gene Expression, Cellular Assembly and Organization	41
	Cellular Assembly and Organization, Cellular Function and Maintenance, Protein Synthesis	41
	Gene Expression, Cell Cycle, Cell-To-Cell Signaling and Interaction	35
	DNA Replication, Recombination, and Repair, Nucleic Acid Metabolism, Small Molecule Biochemistry	33
TCDD	Post-Translational Modification, Genetic Disorder, Hematological Disease	49
	Cell Cycle, Cellular Assembly and Organization, DNA Replication, Recombination, and Repair	47
	Cellular Assembly and Organization, DNA Replication, Recombination, and Repair, Decreased Levels of Albumin	45
	DNA Replication, Recombination, and Repair, Energy Production, Nucleic Acid Metabolism	44
	DNA Replication, Recombination, and Repair, Cell Cycle, Cellular Assembly and Organization	37

**Table 3:** Top canonical pathways for genes differentially expressed in response to BPA, E2 and TCDD identified by IPA.

Chemical	Top canonical pathway	P-Value
BPA	RAN Signaling	5.31E-02
	Endoplasmic Reticulum Stress Pathway	6.34E-02
	Leukocyte Extravasation Signaling	1.24E-01
	Retinoic acid Mediated Apoptosis Signaling	1.54E-01
	Colorectal Cancer Metastasis Signaling	1.93E-01
E2	Cell Cycle: G1/S Checkpoint Regulation	1.01E-03
	PI3K/AKT Signaling	1.52E-03
	Role of NFAT in Regulation of the Immune Response	1.83E-03
	p53 Signaling	3.46E-03
	Aryl Hydrocarbon Receptor Signaling	3.63E-03
TCDD	Cell Cycle Control of Chromosomal Replication	1.20E-09
	Role of BRCA1 in DNA Damge Response	1.72E-07
	Mismatch Repair in Eukaryotes	2.47E-05
	Hereditary Breast Cancer Signaling	9.45E-04
	Role of CHK Proteins in Cell Cycle Checkpoint Control	1.00E-02

In addition, a list of top KEGG pathways affected by BPA, E2 and TCDD identified by Pathway Express was summarized in Table S2. By inputting the list of genes significantly differentially expressed in response to BPA, E2 and TCDD into Pathway Express, 12 KEGG pathways, but without statistical significance, were found to be affected by BPA, while 27 and 9 KEGG pathways were found to be significantly affected by E2 and TCDD, respectively. As an example, “Pathways in cancer” of KEGG mapped with genes significantly differentially expressed in response to BPA, E2 and TCDD using KegArray was illustrated in Figure S4.

### Validation by real-time PCR

**Figure 3:**
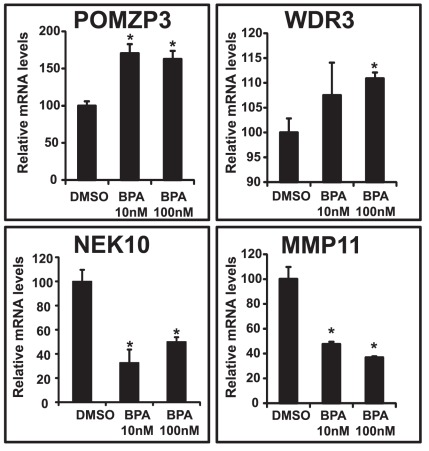
Validation of POMZP1, WDR3, NEK10 and MMP11 expression. Cells were treated with BPA at 10 nM and 100 nM for 24 h, and then the expression of POMZP1, WDR3, NEK10 and MMP11 was examined by real-time PCR. *P<0.05 vs. DMSO control cells.

To validate the microarray data and to identify potential biomarkers for BPA toxicity in hFFCs derived from HS patients, the expression of the most up- or down-regulated genes (POMZP3, 1.46-fold; WDR3, 1.45-fold; NEK10, 0.44-fold; MMP11, 0.41-fold) in response to BPA was validated by real-time PCR. As the results show in Figure 3, the PCR data showed good concordance with the microarray data in terms of the expression direction (up- or down-regulation). A significant increase in the mRNA levels of POMZP3 and WDR3 and a significant decrease in the mRNA levels of NEK10 and MMP11 were observed following BPA treatments at high and/or low concentrations (10 nM and 100 nM, respectively).

### Comparison of MMP11 expression levels in hFFCs derived from child HS and CO patients

**Figure 4:**
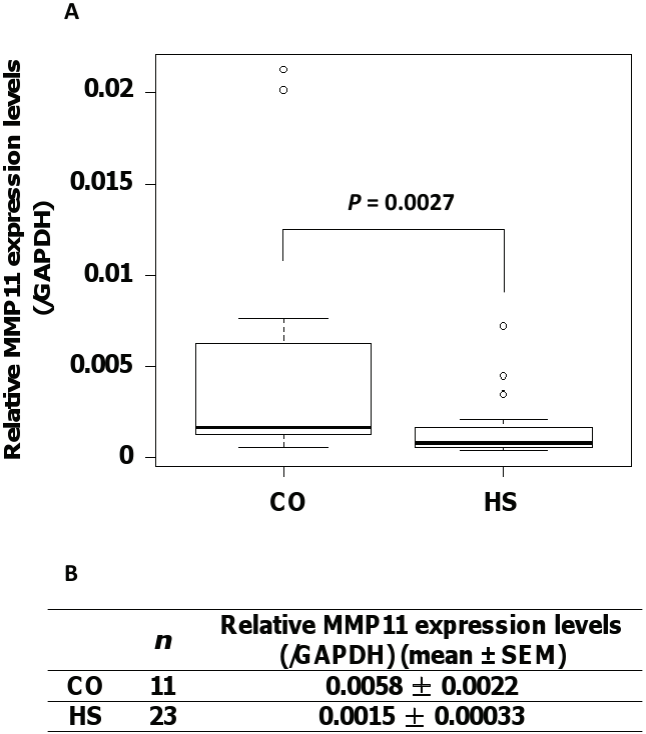
Reduced levels of MMP11 expression in hFFCs derived from child HS patients. Significantly lower MMP11 expression was observed in hFFCs derived from the HS (*n* = 23) group compared with the CO (*n* = 11) group by TaqMan real-time PCR. (***A***) Boxplot and (***B***) summary of the quantitative data comparing MMP11 expression levels in HS and CO groups.

To further investigate the potential role of MMP11 in the development of HS, we examined the expression levels of MMP11 in hFFCs derived from child HS and CO patients (*n* = 23 and 11, respectively). As shown Figure 4, the mean MMP11 expression level, normalized to GAPDH, in the HS group was 0.0015 and in the CO group, 0.0058. Significantly lower MMP11 expression levels were observed in the HS group compared with the CO group (0.25-fold, *P* = 0.0027).

## Discussion

To better understand the molecular basis of the effects of BPA on human reproductive health, target genes of low-dose BPA exposure were identified in hFFCs derived from child HS patients using DNA microarray analysis. Human foreskin tissues obtained from patients with HS have been used as *in vitro* models to define the etiology of HS [20]–[22]. However, these investigations have not delineated the relative contribution of environmental factors. To our knowledge, our study is the first report to use hFFCs to investigate the potential effects of BPA on the development of HS. The concentration of BPA used to treat the cells in our microarray analysis was 10 nM, which is below the dose of 50 µg/kg/day (approximately 200 nM for *in vitro* cell or organ culture studies) usually considered as safe for humans [7]. Moreover, this dose is in the concentration range of 1–19.4 nM that is commonly detected in human tissues and fluids [4].

In this study, we compared the gene expression profiles of hFFCs in response to BPA, E2 and TCDD. Using PCA, we found that the effect of BPA is similar to that of E2 according to PC1 but is similar to that of TCDD according to PC2. Forty-three genes were found to be affected exclusively by BPA, underscoring the concept that the effects observed are ER and AhR-independent (Figure 1). In our previous study, we examined the estrogenic activity of BPA in estrogen receptor 1 (ESR1)-positive BG1Luc4E2 human ovarian cancer cells and found that BPA increased the ESR1-induced luciferase activity in a dose-dependent manner with a lowest observed effect at 100 nM [23]. Although differences exist between cell lines, it is possible that the underlying mechanisms of the endocrine-disrupting effects of BPA at doses lower than the reference limits might involve pathways other than estrogen signaling. Indeed, differences in transcript profiles in response to BPA and E2 have been previously described in ESR1-positive human cells [24]. Furthermore, amore recent study reported that BPA might lead to severe malformation during vertebrate embryogenesis, while no effects were seen with exposure to the E2 or ER-antagonist ICI 182,780 [25].

It is not unexpected that the largest biological network identified by IPA analysis with genes differentially expressed in response to BPA was entitled “Endocrine System Disorders, Gastrointestinal Disease, Genetic Disorder” (Table 2 and Figure 2A). It should be noted that this network contains three genes (ZNF222, ZNF224 and ZNF461) that belong to the zinc finger protein (ZFP) family. ZFPs are among the most abundant proteins in eukaryotic genomes and play various roles in the regulation of transcription [26]. The biological function of ZNF222 and ZNF461 remains to be investigated, but ZNF224 participates in key cellular processes, such as regulation of cell growth [27]. Previous reports have revealed that ZNF224 might play a critical role in bladder carcinogenesis by regulating the apoptosis of bladder cancer cells [28]. None of these three ZNFs have been previously associated with the development of HS. However, two other zinc finger box genes, ZEB1 and ZEB2, have been associated with HS [20], [29]. Our data indicate that ZFP-mediated transcriptional activity might be required for the effect of BPA on human reproduction. It is known that zinc finger structures are as diverse as their functions [26]. Therefore, it is likely that further investigations into the function of ZFPs in transcriptional regulation will provide novel insights to explain the association we found between ZFP expression and low-dose BPA exposure regarding the pathogenesis of HS.

The expression of four of the significantly differentially expressed genes identified in the microarray analysis was verified by real-time PCR analysis. Of particular interest, MMP11 (0.47-fold and 0.37-fold at 10 nM and 100 nM, respectively), which is involved in the “Cell Death, Cellular Growth and Proliferation, Cancer” network, was shown to be down-regulated (Figures 2B and 3). The matrix metalloproteinases (MMPs) are zinc-dependent endopeptidases that are involved in the breakdown of extracellular matrix (ECM) in normal physiological processes, such as embryonic development, reproduction, and tissue remodeling, as well as in disease processes, such as arthritis and metastasis [30], [31]. It is well known that MMP11 is overexpressed in several human cancers, including breast, cervix, colon, ovary, prostate, and stomach cancers [30], [32]–[34]. Several MMPs have been implicated in ECM degradation associated with tumor growth and angiogenesis, which is required for a cancer cell to invade a nearby blood vessel (intravasation) and then to extravasate at a distant location and invade the distant tissue in order to seed a new metastatic site [35].

To our knowledge, there have not been any reports of human congenital genital disorders associated with MMP11. However, it has been reported that MMPs play a critical role in cell fate and behavior during many developmental processes [31], [36]. Both genetic analysis using transgenic mice and pharmacogenetic studies with chemical inhibitors have elucidated that loss of function of MMPs, in particular MMP11, might induce dysregulation in cell migration and apoptosis during tissue remodeling or branching of mammary epithelial cells [37], [38]. A more recent study in the model insect, *Tribolium*, explored MMP functions *in vivo* and found that knockdown of MMPs using genetic interference resulted in malformation in tracheal and gut development during beetle embryogenesis and pupal morphogenesis [39]. It is known that epithelial seam formation and remodeling during urethral formation play important roles in the etiology of HS. The urethral abnormalities seen in HS can be viewed as a failure of epithelial cell adhesion [40]. Therefore, we hypothesized that downregulation of MMP11 expression might decrease cellular adhesion in the developing male urethra and ventral penile skin, which might result in the abortive penile development seen in HS.

To further confirm this hypothesis, we compared the expression levels of MMP11 in hFFCs derived from child HS and CO patients (*n* = 23 and 11, respectively). In 2001, Skakkebaek and his colleagues proposed a concept of TDS: impaired development of fetal testes could lead to increased risks of CO, HS, decreased spermatogenesis or testicular cancer [2]. However, they have recently changed their opinion and now suggest that HS is only marginally associated with TDS [3]. Although much remains to be determined, it is likely that the molecular etiology of HS and CO is different. CO is the absence of one or both testes from the scrotum and is the most common congenital abnormality in boys with a reported prevalence at birth of approximately 2–9%, according to registry data [41]. Impaired descent of the testes is thought to be fetal in origin, and if the *in utero* development of the testicles is impaired then their production of insulin-like factor 3 and especially testosterone may be reduced, which may lead to some degree of CO [3], [42]. However, it is likely that isolated HS may have a different etiological mechanism, including a congenital developmental problem restricted to the penis [43]. Rey *et al.* found that most boys (85%) with isolated HS had, in general, normal testicular endocrinology in contrast to those with HS combined with other genital abnormalities [44]. In this study, only child HS and CO patients without other genital malformations of syndromes were recruited. Therefore, hFFCs derived from foreskin tissues of child CO patients might be viewed as the control group in this study. We found that MMP11 expression in the HS group was significantly lower than in the CO group (0.25-fold, *P* = 0.0027) (Figure 4). This result is in accordance with our hypothesis that downregulation of MMP11 expression might be related with the pathology of HS. Although the urethral tissue was not directly examined, it is possible that there is also a potential effect of MMP11 on urethral development.

In summary, the present study examined targets of low-dose BPA exposure and transcriptome differences in response to BPA, E2 and TCDD in hFFCs derived from child HS patients using DNA microarray analysis. Of particular interest, the expression of MMP11 was found to be downregulated by BPA in a dose-dependent manner. Furthermore, we also found that MMP11 expression in the HS group was significantly lower than in the CO group. Our findings suggested that the involvement of BPA in the development of HS might relate to downregulation of MMP11 expression. Further study of the novel target genes identified in this study during genital tubercle development might increase our knowledge of the molecular basis of the effects of BPA on human reproductive health.

## Supporting Information

Figure S1 **Red indicates upregulated genes, green indicates downregulated genes, and white indicates genes that were not annotated in this array but form part of this network.** The bottom numbers indicate the fold changes induced by BPA and the top numbers is the P-values between DMSO control group and BPA treated group. (***A***) “Cellular Growth and Proliferation, Hematological System Development and Function, Cellular Development” network; (***B***) “Cellular Assembly and Organization, Cellular Function and Maintenance, Cell Cycle” network.
(DOCX)

Figure S2 **Red indicates upregulated genes, green indicates downregulated genes, and white indicates genes that were not annotated in this array but form part of this network.** The bottom numbers indicate the fold changes induced by E2 and the top numbers is the P-values between DMSO control group and E2 treated group. (***A***) “Cellular Growth and Proliferation, Skeletal and Muscular System Development and Function, Cell Cycle” network; (***B***) “DNA Replication, Recombination, and Repair, Gene Expression, Cellular Assembly and Organization” network; (***C***) “Cellular Assembly and Organization, Cellular Function and Maintenance, Protein Synthesis” network; (***D***) “Gene Expression, Cell Cycle, Cell-To-Cell Signaling and Interaction” network; (***E***) “DNA Replication, Recombination, and Repair, Nucleic Acid Metabolism, Small Molecule Biochemistry” network.
(DOCX)

Figure S3 **Red indicates upregulated genes, green indicates downregulated genes, and white indicates genes that were not annotated in this array but form part of this network.** The bottom numbers indicate the fold changes induced by TCDD and the top numbers is the P-values between DMSO control group and TCDD treated group. (***A***) “Post-Translational Modification, Genetic Disorder, Hematological Disease” network; (***B***) “Cell Cycle, Cellular Assembly and Organization, DNA Replication, Recombination, and Repair” network; (***C***) “Cellular Assembly and Organization, DNA Replication, Recombination, and Repair, Decreased Levels of Albumin” network; (***D***) “DNA Replication, Recombination, and Repair, Energy Production, Nucleic Acid Metabolism” network; (***E***) “DNA Replication, Recombination, and Repair, Cell Cycle, Cellular Assembly and Organization” network.
(DOCX)

Figure S4 **“Pathways in cancer” of KEGG was mapped with genes significantly differentially expressed in response to BPA (** ***A*** **), E2 (** ***B*** **) and TCDD (** ***C*** **).**
(DOCX)

Table S1 **Comparison of the gene expression profiles of hFFCs in response to BPA, E2 and TCDD.**
(DOCX)

Table S2 **KEGG pathways affected by BPA, E2 and TCDD identified by Pathway Express.**
(DOCX)
