# Supplementary figures and images for: Identification of Novel Low-Dose Bisphenol A Targets in Human Foreskin Fibroblast Cells Derived from Hypospadias Patients
Source: PLoS One. 2012 May 4;7(5):e36711. doi: 10.1371/journal.pone.0036711 (PMC3344929; doi:10.1371/journal.pone.0036711)

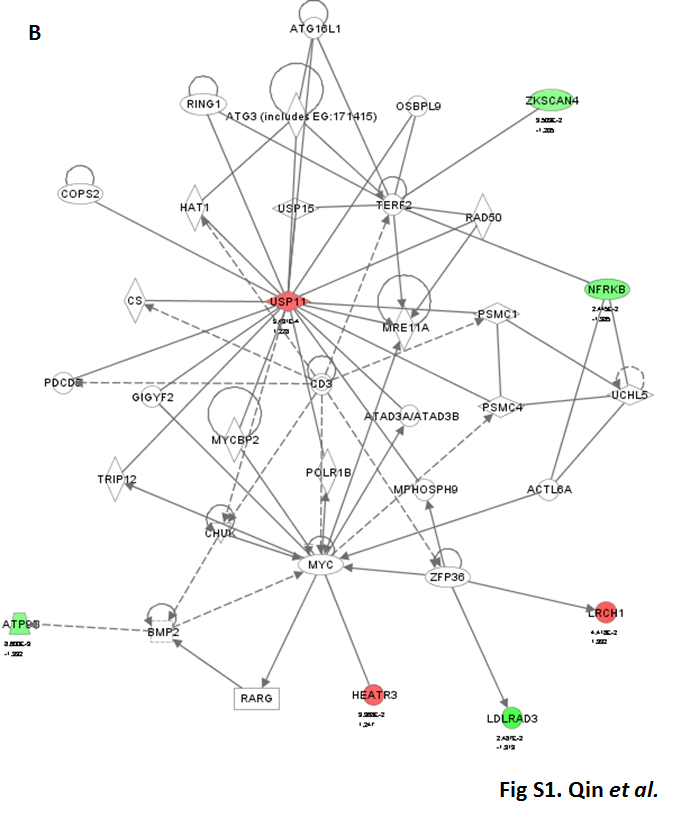

Supplement: Figure S1 — Red indicates upregulated genes, green indicates downregulated genes, and white indicates genes that were not annotated in this array but form part of this network. The bottom numbers indicate the fold changes induced by BPA and the top numbers is the P-values between DMSO control group and BPA treated group. (A) “Cellular Growth and Proliferation, Hematological System Development and Function, Cellular Development” network; (B) “Cellular Assembly and Organization, Cellular Function and Maintenance, Cell Cycle” network. (DOCX) [file pone.0036711.s001.docx]
