# Supplementary material for: Identification of Novel Low-Dose Bisphenol A Targets in Human Foreskin Fibroblast Cells Derived from Hypospadias Patients
Source: PLoS One. 2012 May 4;7(5):e36711. doi: 10.1371/journal.pone.0036711 (PMC3344929; doi:10.1371/journal.pone.0036711)
Supplement: Table S1 — Comparison of the gene expression profiles of hFFCs in response to BPA, E2 and TCDD. (DOCX) [file pone.0036711.s005.docx]

| **Gene Symbol** | **Gene Name** |
| --- | --- |
| ***BPA specific (n = 43)*** |  |
| ABCA7 | ATP-binding cassette, sub-family A (ABC1), member 7 |
| ACER2 | alkaline ceramidase 2 |
| ARHGAP21 | Rho GTPase activating protein 21 |
| ATP9B | ATPase, class II, type 9B |
| C2orf81 | chromosome 2 open reading frame 81 |
| CNTLN | centlein, centrosomal protein |
| EIF2AK3 | eukaryotic translation initiation factor 2-alpha kinase 3 |
| EIF4ENIF1 | eukaryotic translation initiation factor 4E nuclear import factor 1 |
| EPB41L4A | erythrocyte membrane protein band 4.1 like 4A |
| EXD2 | exonuclease 3'-5' domain containing 2 |
| EXD3 | exonuclease 3'-5' domain containing 3 |
| FAM22F | family with sequence similarity 22, member F |
| FBXO18 | F-box protein, helicase, 18 |
| FKBP4 | FK506 binding protein 4, 59kDa |
| FLJ45244 | hypothetical locus FLJ45244 |
| FSIP1 | fibrous sheath interacting protein 1 |
| KLRAQ1 | KLRAQ motif containing 1 |
| KPNA5 | karyopherin alpha 5 (importin alpha 6) |
| LGALS7 | lectin, galactoside-binding, soluble, 7 |
| LOC100128714 | hypothetical protein LOC100128714 |
| LOC100144603 | hypothetical transcript |
| LOC100144603 | hypothetical transcript |
| LOC284933 | hypothetical protein LOC284933 |
| LRRC42 | leucine rich repeat containing 42 |
| MDM1 | Mdm1 nuclear protein homolog (mouse) |
| MPP1 | membrane protein, palmitoylated 1, 55kDa |
| NUDCD1 | NudC domain containing 1 |
| POMZP3 | POM (POM121 homolog, rat) and ZP3 fusion |
| RAPGEF3 | Rap guanine nucleotide exchange factor (GEF) 3 |
| SCARNA9L | small Cajal body-specific RNA 9-like (retrotransposed) |
| SPIN4 | spindlin family, member 4 |
| TMEM190 | transmembrane protein 190 |
| TNFRSF10C | tumor necrosis factor receptor superfamily, member 10c, decoy without an intracellular domain |
| TXNL4B | thioredoxin-like 4B |
| WDR3 | WD repeat domain 3 |
| WNT3A | wingless-type MMTV integration site family, member 3A |
| ZBTB25 | zinc finger and BTB domain containing 25 |
| ZKSCAN4 | zinc finger with KRAB and SCAN domains 4 |
| ZNF222 | zinc finger protein 222 |
| ZNF223 | zinc finger protein 223 |
| ZNF649 | zinc finger protein 649 |
| ZNF791 | zinc finger protein 791 |
| ZNF850P | zinc finger protein 850 (pseudogene) |
| ***E2 specific (n = 628)*** |  |
| 41157 | septin 5 |
| 41160 | septin 8 |
| ACTBL2 | actin, beta-like 2 |
| ACYP1 | acylphosphatase 1, erythrocyte (common) type |
| ADHFE1 | alcohol dehydrogenase, iron containing, 1 |
| ADK | adenosine kinase |
| ADM | adrenomedullin |
| ADPRHL2 | ADP-ribosylhydrolase like 2 |
| AGFG1 | ArfGAP with FG repeats 1 |
| AKAP1 | A kinase (PRKA) anchor protein 1 |
| AKAP11 | A kinase (PRKA) anchor protein 11 |
| AKT2 | v-akt murine thymoma viral oncogene homolog 2 |
| ALDH3B1 | aldehyde dehydrogenase 3 family, member B1 |
| ALS2 | amyotrophic lateral sclerosis 2 (juvenile) |
| AMOTL1 | angiomotin like 1 |
| ANKMY2 | ankyrin repeat and MYND domain containing 2 |
| ANPEP | alanyl (membrane) aminopeptidase |
| ANTXR2 | anthrax toxin receptor 2 |
| ARHGAP26 | Rho GTPase activating protein 26 |
| ARHGEF17 | Rho guanine nucleotide exchange factor (GEF) 17 |
| ARID4B | AT rich interactive domain 4B (RBP1-like) |
| ARID5B | AT rich interactive domain 5B (MRF1-like) |
| ARL6 | ADP-ribosylation factor-like 6 |
| ARVCF | armadillo repeat gene deletes in velocardiofacial syndrome |
| ASAM | adipocyte-specific adhesion molecule |
| ASMTL | acetylserotonin O-methyltransferase-like |
| ASPH | aspartate beta-hydroxylase |
| ASPN | asporin |
| ASPN | asporin |
| ASTN2 | astrotactin 2 |
| ATE1 | arginyltransferase 1 |
| ATP8B1 | ATPase, class I, type 8B, member 1 |
| AUP1 | ancient ubiquitous protein 1 |
| AXIN2 | axin 2 |
| B3GNT2 | UDP-GlcNAc:betaGal beta-1,3-N-acetylglucosaminyltransferase 2 |
| BAG4 | BCL2-associated athanogene 4 |
| BAT4 | HLA-B associated transcript 4 |
| BCORL2 | BCL6 co-repressor-like 2 |
| BCR | breakpoint cluster region |
| BCR | breakpoint cluster region |
| BICD2 | bicaudal D homolog 2 (Drosophila) |
| BIVM | basic, immunoglobulin-like variable motif containing |
| BLZF1 | basic leucine zipper nuclear factor 1 |
| BMP4 | bone morphogenetic protein 4 |
| BRIX1 | BRX1, biogenesis of ribosomes, homolog (S. cerevisiae) |
| BZRAP1 | benzodiazapine receptor (peripheral) associated protein 1 |
| BZW1 | basic leucine zipper and W2 domains 1 |
| C10orf104 | chromosome 10 open reading frame 104 |
| C10orf25 | chromosome 10 open reading frame 25 |
| C10orf28 | chromosome 10 open reading frame 28 |
| C11orf57 | chromosome 11 open reading frame 57 |
| C11orf63 | chromosome 11 open reading frame 63 |
| C12orf70 | chromosome 12 open reading frame 70 |
| C13orf27 | chromosome 13 open reading frame 27 |
| C14orf159 | chromosome 14 open reading frame 159 |
| C14orf167 | chromosome 14 open reading frame 167 |
| C14orf45 | chromosome 14 open reading frame 45 |
| C14orf49 | chromosome 14 open reading frame 49 |
| C15orf5 | chromosome 15 open reading frame 5 |
| C17orf69 | chromosome 17 open reading frame 69 |
| C19orf73 | chromosome 19 open reading frame 73 |
| C1orf103 | chromosome 1 open reading frame 103 |
| C1orf167 | chromosome 1 open reading frame 167 |
| C1orf53 | chromosome 1 open reading frame 53 |
| C1orf9 | chromosome 1 open reading frame 9 |
| C1orf95 | chromosome 1 open reading frame 95 |
| C1RL | complement component 1, r subcomponent-like |
| C20orf12 | chromosome 20 open reading frame 12 |
| C21orf2 | chromosome 21 open reading frame 2 |
| C21orf2 | chromosome 21 open reading frame 2 |
| C21orf59 | chromosome 21 open reading frame 59 |
| C22orf46 | chromosome 22 open reading frame 46 |
| C2CD2L | C2CD2-like |
| C3orf47 | chromosome 3 open reading frame 47 |
| C3orf71 | chromosome 3 open reading frame 71 |
| C4orf41 | chromosome 4 open reading frame 41 |
| C5orf13 | chromosome 5 open reading frame 13 |
| C5orf27 | chromosome 5 open reading frame 27 |
| C5orf62 | chromosome 5 open reading frame 62 |
| C6orf1 | chromosome 6 open reading frame 1 |
| C7orf40 | chromosome 7 open reading frame 40 |
| C7orf60 | chromosome 7 open reading frame 60 |
| CABC1 | chaperone, ABC1 activity of bc1 complex homolog (S. pombe) |
| CACNA2D4 | calcium channel, voltage-dependent, alpha 2/delta subunit 4 |
| CAMSAP1 | calmodulin regulated spectrin-associated protein 1 |
| CAPRIN1 | cell cycle associated protein 1 |
| CARD6 | caspase recruitment domain family, member 6 |
| CASP4 | caspase 4, apoptosis-related cysteine peptidase |
| CASP5 | caspase 5, apoptosis-related cysteine peptidase |
| CCDC115 | coiled-coil domain containing 115 |
| CCDC115 | coiled-coil domain containing 115 |
| CCDC149 | coiled-coil domain containing 149 |
| CCDC80 | coiled-coil domain containing 80 |
| CCND1 | cyclin D1 |
| CCND3 | cyclin D3 |
| CCNG2 | cyclin G2 |
| CD86 | CD86 molecule |
| CDC14C | CDC14 cell division cycle 14 homolog C (S. cerevisiae) |
| CDK13 | cyclin-dependent kinase 13 |
| CEP170 | centrosomal protein 170kDa |
| CEP170 | centrosomal protein 170kDa |
| CHUK | conserved helix-loop-helix ubiquitous kinase |
| CIRBP | cold inducible RNA binding protein |
| CITED2 | Cbp/p300-interacting transactivator, with Glu/Asp-rich carboxy-terminal domain, 2 |
| CITED4 | Cbp/p300-interacting transactivator, with Glu/Asp-rich carboxy-terminal domain, 4 |
| CLIP4 | CAP-GLY domain containing linker protein family, member 4 |
| CMTM4 | CKLF-like MARVEL transmembrane domain containing 4 |
| CMTM7 | CKLF-like MARVEL transmembrane domain containing 7 |
| CNKSR3 | CNKSR family member 3 |
| CNN3 | calponin 3, acidic |
| COL11A1 | collagen, type XI, alpha 1 |
| COL5A2 | collagen, type V, alpha 2 |
| COMMD8 | COMM domain containing 8 |
| COPZ2 | coatomer protein complex, subunit zeta 2 |
| CORO6 | coronin 6 |
| CP110 | CP110 protein |
| CRCP | CGRP receptor component |
| CSF3 | colony stimulating factor 3 (granulocyte) |
| CSNK1E | casein kinase 1, epsilon |
| CSRP2 | cysteine and glycine-rich protein 2 |
| CST6 | cystatin E/M |
| CTDSP2 | CTD (carboxy-terminal domain, RNA polymerase II, polypeptide A) small phosphatase 2 |
| CXCL12 | chemokine (C-X-C motif) ligand 12 (stromal cell-derived factor 1) |
| CXCL2 | chemokine (C-X-C motif) ligand 2 |
| CXCL3 | chemokine (C-X-C motif) ligand 3 |
| CXorf15 | chromosome X open reading frame 15 |
| CXXC5 | CXXC finger 5 |
| CYR61 | cysteine-rich, angiogenic inducer, 61 |
| DAB1 | disabled homolog 1 (Drosophila) |
| DAB2 | disabled homolog 2, mitogen-responsive phosphoprotein (Drosophila) |
| DCAF8 | DDB1 and CUL4 associated factor 8 |
| DCTPP1 | dCTP pyrophosphatase 1 |
| DDAH1 | dimethylarginine dimethylaminohydrolase 1 |
| DDAH2 | dimethylarginine dimethylaminohydrolase 2 |
| DDX10 | DEAD (Asp-Glu-Ala-Asp) box polypeptide 10 |
| DKFZp667E0512 | hypothetical protein DKFZp667E0512 |
| DLC1 | deleted in liver cancer 1 |
| DLL3 | delta-like 3 (Drosophila) |
| DNAJC3 | DnaJ (Hsp40) homolog, subfamily C, member 3 |
| DNAJC5 | DnaJ (Hsp40) homolog, subfamily C, member 5 |
| DNAL4 | dynein, axonemal, light chain 4 |
| DNMBP | dynamin binding protein |
| DOCK7 | dedicator of cytokinesis 7 |
| DPP9 | dipeptidyl-peptidase 9 |
| DPP9 | dipeptidyl-peptidase 9 |
| DSE | dermatan sulfate epimerase |
| DVL1 | dishevelled, dsh homolog 1 (Drosophila) |
| DYNC2LI1 | dynein, cytoplasmic 2, light intermediate chain 1 |
| DYRK4 | dual-specificity tyrosine-(Y)-phosphorylation regulated kinase 4 |
| DYX1C1 | dyslexia susceptibility 1 candidate 1 |
| EDEM3 | ER degradation enhancer, mannosidase alpha-like 3 |
| EEPD1 | endonuclease/exonuclease/phosphatase family domain containing 1 |
| EFNA4 | ephrin-A4 |
| EHBP1 | EH domain binding protein 1 |
| EHMT1 | euchromatic histone-lysine N-methyltransferase 1 |
| EIF1AD | eukaryotic translation initiation factor 1A domain containing |
| EIF2B3 | eukaryotic translation initiation factor 2B, subunit 3 gamma, 58kDa |
| EIF2C1 | eukaryotic translation initiation factor 2C, 1 |
| EIF3F | eukaryotic translation initiation factor 3, subunit F |
| ELOVL1 | elongation of very long chain fatty acids (FEN1/Elo2, SUR4/Elo3, yeast)-like 1 |
| EMP1 | epithelial membrane protein 1 |
| ENAH | enabled homolog (Drosophila) |
| EPOR | erythropoietin receptor |
| ESRRA | estrogen-related receptor alpha |
| ESYT3 | extended synaptotagmin-like protein 3 |
| ETAA1 | Ewing tumor-associated antigen 1 |
| ETHE1 | ethylmalonic encephalopathy 1 |
| EVC2 | Ellis van Creveld syndrome 2 |
| EVPLL | envoplakin-like |
| EXD3 | exonuclease 3'-5' domain containing 3 |
| FADD | Fas (TNFRSF6)-associated via death domain |
| FADS2 | fatty acid desaturase 2 |
| FAM107B | family with sequence similarity 107, member B |
| FAM151B | family with sequence similarity 151, member B |
| FAM162B | family with sequence similarity 162, member B |
| FAM199X | family with sequence similarity 199, X-linked |
| FAM40B | family with sequence similarity 40, member B |
| FAM86B1 | family with sequence similarity 86, member B1 |
| FARSA | phenylalanyl-tRNA synthetase, alpha subunit |
| FAT3 | FAT tumor suppressor homolog 3 (Drosophila) |
| FBLL1 | fibrillarin-like 1 |
| FBN2 | fibrillin 2 |
| FBXO11 | F-box protein 11 |
| FBXO30 | F-box protein 30 |
| FBXO34 | F-box protein 34 |
| FBXO6 | F-box protein 6 |
| FCHSD1 | FCH and double SH3 domains 1 |
| FCRLB | Fc receptor-like B |
| FEM1B | fem-1 homolog b (C. elegans) |
| FIGN | fidgetin |
| FLJ31813 | hypothetical protein FLJ31813 |
| FLJ35220 | hypothetical protein FLJ35220 |
| FLJ39609 | similar to hCG1995469 |
| FLJ41484 | hypothetical LOC650669 |
| FLJ42709 | hypothetical LOC441094 |
| FMNL2 | formin-like 2 |
| FOS | FBJ murine osteosarcoma viral oncogene homolog |
| FOXI2 | forkhead box I2 |
| FOXK2 | forkhead box K2 |
| FOXP1 | forkhead box P1 |
| FRY | furry homolog (Drosophila) |
| FTL | ferritin, light polypeptide |
| FUT8 | fucosyltransferase 8 (alpha (1,6) fucosyltransferase) |
| GADD45A | growth arrest and DNA-damage-inducible, alpha |
| GADD45G | growth arrest and DNA-damage-inducible, gamma |
| GAL | galanin prepropeptide |
| GAS2L1 | growth arrest-specific 2 like 1 |
| GATAD2A | GATA zinc finger domain containing 2A |
| GFOD2 | glucose-fructose oxidoreductase domain containing 2 |
| GK | glycerol kinase |
| GLI2 | GLI family zinc finger 2 |
| GLS | glutaminase |
| GLT8D2 | glycosyltransferase 8 domain containing 2 |
| GLTSCR1 | glioma tumor suppressor candidate region gene 1 |
| GLTSCR2 | glioma tumor suppressor candidate region gene 2 |
| GNG11 | guanine nucleotide binding protein (G protein), gamma 11 |
| GNG8 | guanine nucleotide binding protein (G protein), gamma 8 |
| GNL2 | guanine nucleotide binding protein-like 2 (nucleolar) |
| GPATCH4 | G patch domain containing 4 |
| GPC1 | glypican 1 |
| GPR124 | G protein-coupled receptor 124 |
| GPR125 | G protein-coupled receptor 125 |
| GPR172A | G protein-coupled receptor 172A |
| GREM1 | gremlin 1, cysteine knot superfamily, homolog (Xenopus laevis) |
| GRIK2 | glutamate receptor, ionotropic, kainate 2 |
| GRIN3B | glutamate receptor, ionotropic, N-methyl-D-aspartate 3B |
| GSK3B | glycogen synthase kinase 3 beta |
| GSN | gelsolin (amyloidosis, Finnish type) |
| GSN | gelsolin (amyloidosis, Finnish type) |
| GSTO2 | glutathione S-transferase omega 2 |
| GTPBP3 | GTP binding protein 3 (mitochondrial) |
| H2AFV | H2A histone family, member V |
| H6PD | hexose-6-phosphate dehydrogenase (glucose 1-dehydrogenase) |
| HAUS2 | HAUS augmin-like complex, subunit 2 |
| HAUS3 | HAUS augmin-like complex, subunit 3 |
| HDAC11 | histone deacetylase 11 |
| HDAC7 | histone deacetylase 7 |
| HDAC8 | histone deacetylase 8 |
| HIPK1 | homeodomain interacting protein kinase 1 |
| HIST1H2AE | histone cluster 1, H2ae |
| HIST1H2BC | histone cluster 1, H2bc |
| HIST1H2BD | histone cluster 1, H2bd |
| HIST1H2BH | histone cluster 1, H2bh |
| HIST1H2BI | histone cluster 1, H2bi |
| HIST1H2BK | histone cluster 1, H2bk |
| HIST1H2BL | histone cluster 1, H2bl |
| HIST1H2BM | histone cluster 1, H2bm |
| HIST1H2BO | histone cluster 1, H2bo |
| HIVEP3 | human immunodeficiency virus type I enhancer binding protein 3 |
| HMGN5 | high-mobility group nucleosome binding domain 5 |
| HMX2 | H6 family homeobox 2 |
| HOPX | HOP homeobox |
| HOXD9 | homeobox D9 |
| HPCAL1 | hippocalcin-like 1 |
| HSP90AB1 | heat shock protein 90kDa alpha (cytosolic), class B member 1 |
| HSP90AB5P | heat shock protein 90kDa alpha (cytosolic), class B member 5 (pseudogene) |
| HSPA8 | heat shock 70kDa protein 8 |
| HSPB2 | heat shock 27kDa protein 2 |
| HTRA2 | HtrA serine peptidase 2 |
| IFI16 | interferon, gamma-inducible protein 16 |
| IFT52 | intraflagellar transport 52 homolog (Chlamydomonas) |
| IFT74 | intraflagellar transport 74 homolog (Chlamydomonas) |
| IFT81 | intraflagellar transport 81 homolog (Chlamydomonas) |
| IGBP1 | immunoglobulin (CD79A) binding protein 1 |
| IL18 | interleukin 18 (interferon-gamma-inducing factor) |
| IL4R | interleukin 4 receptor |
| IL8 | interleukin 8 |
| IMP3 | IMP3, U3 small nucleolar ribonucleoprotein, homolog (yeast) |
| IP6K2 | inositol hexakisphosphate kinase 2 |
| ISYNA1 | inositol-3-phosphate synthase 1 |
| ITGA2 | integrin, alpha 2 (CD49B, alpha 2 subunit of VLA-2 receptor) |
| ITGB8 | integrin, beta 8 |
| ITPR1 | inositol 1,4,5-triphosphate receptor, type 1 |
| KCNQ5 | potassium voltage-gated channel, KQT-like subfamily, member 5 |
| KIAA0427 | KIAA0427 |
| KIAA1274 | KIAA1274 |
| KIAA1407 | KIAA1407 |
| KILLIN | killin protein |
| KLC2 | kinesin light chain 2 |
| KLC2 | kinesin light chain 2 |
| KLHL18 | kelch-like 18 (Drosophila) |
| KLHL21 | kelch-like 21 (Drosophila) |
| KRCC1 | lysine-rich coiled-coil 1 |
| KRR1 | KRR1, small subunit (SSU) processome component, homolog (yeast) |
| KRTAP1-3 | keratin associated protein 1-3 |
| KRTAP1-3 | keratin associated protein 1-3 |
| KRTAP20-2 | keratin associated protein 20-2 |
| KRTAP3-1 | keratin associated protein 3-1 |
| L3MBTL | l(3)mbt-like (Drosophila) |
| LACTB | lactamase, beta |
| LARP1B | La ribonucleoprotein domain family, member 1B |
| LARP4 | La ribonucleoprotein domain family, member 4 |
| LASP1 | LIM and SH3 protein 1 |
| LBH | limb bud and heart development homolog (mouse) |
| LIMA1 | LIM domain and actin binding 1 |
| LIPA | lipase A, lysosomal acid, cholesterol esterase |
| LITAF | lipopolysaccharide-induced TNF factor |
| LMCD1 | LIM and cysteine-rich domains 1 |
| LMO2 | LIM domain only 2 (rhombotin-like 1) |
| LMOD1 | leiomodin 1 (smooth muscle) |
| LNP1 | leukemia NUP98 fusion partner 1 |
| LOC100049716 | hypothetical protein LOC100049716 |
| LOC100125556 | family with sequence similarity 86, member A pseudogene |
| LOC100128081 | hypothetical LOC100128081 |
| LOC100128239 | hypothetical LOC100128239 |
| LOC100129034 | hypothetical protein LOC100129034 |
| LOC100129186 | hypothetical LOC100129186 |
| LOC100129397 | hypothetical protein LOC100129397 |
| LOC100129514 | hypothetical LOC100129514 |
| LOC100132541 | similar to glycosyltransferase 8 domain containing 3 |
| LOC100132774 | hypothetical LOC100132774 |
| LOC100132831 | A20-binding inhibitor of NF-kappaB activation 2 pseudogene |
| LOC100133131 | hypothetical protein LOC100133131 |
| LOC100133263 | hypothetical LOC100133263 |
| LOC100287006 | hypothetical protein LOC100287006 |
| LOC148709 | actin pseudogene |
| LOC151162 | hypothetical LOC151162 |
| LOC153546 | hypothetical protein LOC153546 |
| LOC253039 | hypothetical LOC253039 |
| LOC282997 | hypothetical protein LOC282997 |
| LOC284242 | hypothetical protein LOC284242 |
| LOC286367 | FP944 |
| LOC388279 | hypothetical gene supported by AF275804 |
| LOC390282 | similar to hCG2040283 |
| LOC441268 | hypothetical LOC441268 |
| LOC642513 | similar to Potassium channel tetramerisation domain containing 9 |
| LOC643783 | hypothetical LOC643783 |
| LOC645431 | hypothetical LOC645431 |
| LOC646396 | similar to hCG2042704 |
| LOC648740 | ACTB pseudogene |
| LOC730101 | hypothetical LOC730101 |
| LOC730338 | hypothetical LOC730338 |
| LOH12CR2 | loss of heterozygosity, 12, chromosomal region 2 |
| LOH3CR2A | loss of heterozygosity, 3, chromosomal region 2, gene A |
| LRRC27 | leucine rich repeat containing 27 |
| LRRFIP2 | leucine rich repeat (in FLII) interacting protein 2 |
| LRTOMT | leucine rich transmembrane and 0-methyltransferase domain containing |
| LUM | lumican |
| LZTFL1 | leucine zipper transcription factor-like 1 |
| MACF1 | microtubule-actin crosslinking factor 1 |
| MAP2K1 | mitogen-activated protein kinase kinase 1 |
| MAP2K5 | mitogen-activated protein kinase kinase 5 |
| MAP3K8 | mitogen-activated protein kinase kinase kinase 8 |
| MARK3 | MAP/microtubule affinity-regulating kinase 3 |
| MDFIC | MyoD family inhibitor domain containing |
| MDK | midkine (neurite growth-promoting factor 2) |
| MED18 | mediator complex subunit 18 |
| MED23 | mediator complex subunit 23 |
| MEIS2 | Meis homeobox 2 |
| MEX3B | mex-3 homolog B (C. elegans) |
| MFSD5 | major facilitator superfamily domain containing 5 |
| MID1 | midline 1 (Opitz/BBB syndrome) |
| MINA | MYC induced nuclear antigen |
| MKS1 | Meckel syndrome, type 1 |
| MLLT3 | myeloid/lymphoid or mixed-lineage leukemia (trithorax homolog, Drosophila); translocated to, 3 |
| MMAB | methylmalonic aciduria (cobalamin deficiency) cblB type |
| MMP14 | matrix metallopeptidase 14 (membrane-inserted) |
| MREG | melanoregulin |
| MRVI1 | murine retrovirus integration site 1 homolog |
| MSRB2 | methionine sulfoxide reductase B2 |
| MT1A | metallothionein 1A |
| MT1B | metallothionein 1B |
| MT1E | metallothionein 1E |
| MT1H | metallothionein 1H |
| MT1L | metallothionein 1L (gene/pseudogene) |
| MTHFD1L | methylenetetrahydrofolate dehydrogenase (NADP+ dependent) 1-like |
| MTSS1 | metastasis suppressor 1 |
| MYH10 | myosin, heavy chain 10, non-muscle |
| MYO5A | myosin VA (heavy chain 12, myoxin) |
| MYOF | myoferlin |
| NAMPT | nicotinamide phosphoribosyltransferase |
| NAV3 | neuron navigator 3 |
| NBPF3 | neuroblastoma breakpoint family, member 3 |
| NCRNA00087 | non-protein coding RNA 87 |
| NEDD4L | neural precursor cell expressed, developmentally down-regulated 4-like |
| NEDD9 | neural precursor cell expressed, developmentally down-regulated 9 |
| NEIL2 | nei like 2 (E. coli) |
| NEK4 | NIMA (never in mitosis gene a)-related kinase 4 |
| NETO1 | neuropilin (NRP) and tolloid (TLL)-like 1 |
| NFASC | neurofascin homolog (chicken) |
| NFATC4 | nuclear factor of activated T-cells, cytoplasmic, calcineurin-dependent 4 |
| NFE2 | nuclear factor (erythroid-derived 2), 45kDa |
| NFKBIA | nuclear factor of kappa light polypeptide gene enhancer in B-cells inhibitor, alpha |
| NIPAL2 | NIPA-like domain containing 2 |
| NIPSNAP3B | nipsnap homolog 3B (C. elegans) |
| NKX6-2 | NK6 homeobox 2 |
| NLRX1 | NLR family member X1 |
| NNT | nicotinamide nucleotide transhydrogenase |
| NPIPL1 | nuclear pore complex interacting protein-like 1 |
| NPIPL2 | nuclear pore complex interacting protein-like 2 |
| NRIP3 | nuclear receptor interacting protein 3 |
| NTN4 | netrin 4 |
| NUCB1 | nucleobindin 1 |
| NUP43 | nucleoporin 43kDa |
| NXT1 | NTF2-like export factor 1 |
| OBFC1 | oligonucleotide/oligosaccharide-binding fold containing 1 |
| OBFC2A | oligonucleotide/oligosaccharide-binding fold containing 2A |
| OBSL1 | obscurin-like 1 |
| ODC1 | ornithine decarboxylase 1 |
| ODZ2 | odz, odd Oz/ten-m homolog 2 (Drosophila) |
| ODZ2 | odz, odd Oz/ten-m homolog 2 (Drosophila) |
| OLR1 | oxidized low density lipoprotein (lectin-like) receptor 1 |
| OPA3 | optic atrophy 3 (autosomal recessive, with chorea and spastic paraplegia) |
| OR10G2 | olfactory receptor, family 10, subfamily G, member 2 |
| OR7E13P | olfactory receptor, family 7, subfamily E, member 13 pseudogene |
| ORAI1 | ORAI calcium release-activated calcium modulator 1 |
| ORC5L | origin recognition complex, subunit 5-like (yeast) |
| PALLD | palladin, cytoskeletal associated protein |
| PARP16 | poly (ADP-ribose) polymerase family, member 16 |
| PARP3 | poly (ADP-ribose) polymerase family, member 3 |
| PARP4 | poly (ADP-ribose) polymerase family, member 4 |
| PAXIP1 | PAX interacting (with transcription-activation domain) protein 1 |
| PCDH9 | protocadherin 9 |
| PDE5A | phosphodiesterase 5A, cGMP-specific |
| PDK2 | pyruvate dehydrogenase kinase, isozyme 2 |
| PECI | peroxisomal D3,D2-enoyl-CoA isomerase |
| PERP | PERP, TP53 apoptosis effector |
| PFKFB2 | 6-phosphofructo-2-kinase/fructose-2,6-biphosphatase 2 |
| PGRMC2 | progesterone receptor membrane component 2 |
| PHGDH | phosphoglycerate dehydrogenase |
| PHLDA2 | pleckstrin homology-like domain, family A, member 2 |
| PI3 | peptidase inhibitor 3, skin-derived |
| PICALM | phosphatidylinositol binding clathrin assembly protein |
| PID1 | phosphotyrosine interaction domain containing 1 |
| PKD1 | polycystic kidney disease 1 (autosomal dominant) |
| PKIG | protein kinase (cAMP-dependent, catalytic) inhibitor gamma |
| PLAT | plasminogen activator, tissue |
| PLAUR | plasminogen activator, urokinase receptor |
| PLEC | plectin |
| PLEKHN1 | pleckstrin homology domain containing, family N member 1 |
| PLK2 | polo-like kinase 2 (Drosophila) |
| PLXNB2 | plexin B2 |
| PNPLA2 | patatin-like phospholipase domain containing 2 |
| PNRC1 | proline-rich nuclear receptor coactivator 1 |
| POLM | polymerase (DNA directed), mu |
| PPM1F | protein phosphatase, Mg2+/Mn2+ dependent, 1F |
| PPM1M | protein phosphatase, Mg2+/Mn2+ dependent, 1M |
| PPP2R2A | protein phosphatase 2 (formerly 2A), regulatory subunit B, alpha isoform |
| PPP3R1 | protein phosphatase 3 (formerly 2B), regulatory subunit B, alpha isoform |
| PQLC3 | PQ loop repeat containing 3 |
| PRAMEF5 | PRAME family member 5 |
| PRDX1 | peroxiredoxin 1 |
| PRNP | prion protein |
| PRPF4 | PRP4 pre-mRNA processing factor 4 homolog (yeast) |
| PRR16 | proline rich 16 |
| PRRT2 | proline-rich transmembrane protein 2 |
| PRRX2 | paired related homeobox 2 |
| PRSS35 | protease, serine, 35 |
| PSORS1C1 | psoriasis susceptibility 1 candidate 1 |
| PSPC1 | paraspeckle component 1 |
| PSTK | phosphoseryl-tRNA kinase |
| PTGS1 | prostaglandin-endoperoxide synthase 1 (prostaglandin G/H synthase and cyclooxygenase) |
| PTP4A1 | protein tyrosine phosphatase type IVA, member 1 |
| PTP4A3 | protein tyrosine phosphatase type IVA, member 3 |
| PTPN1 | protein tyrosine phosphatase, non-receptor type 1 |
| PTPN12 | protein tyrosine phosphatase, non-receptor type 12 |
| PTPN22 | protein tyrosine phosphatase, non-receptor type 22 (lymphoid) |
| PTPRK | protein tyrosine phosphatase, receptor type, K |
| PTPRK | protein tyrosine phosphatase, receptor type, K |
| PTPRS | protein tyrosine phosphatase, receptor type, S |
| PVR | poliovirus receptor |
| PVRL2 | poliovirus receptor-related 2 (herpesvirus entry mediator B) |
| PWP2 | PWP2 periodic tryptophan protein homolog (yeast) |
| PXDN | peroxidasin homolog (Drosophila) |
| PXDN | peroxidasin homolog (Drosophila) |
| PXN | paxillin |
| RAB40AL | RAB40A, member RAS oncogene family-like |
| RABEP2 | rabaptin, RAB GTPase binding effector protein 2 |
| RAMP1 | receptor (G protein-coupled) activity modifying protein 1 |
| RASA3 | RAS p21 protein activator 3 |
| RASSF8 | Ras association (RalGDS/AF-6) domain family (N-terminal) member 8 |
| RBM3 | RNA binding motif (RNP1, RRM) protein 3 |
| RBMS3 | RNA binding motif, single stranded interacting protein |
| RECQL | RecQ protein-like (DNA helicase Q1-like) |
| REP15 | RAB15 effector protein |
| REST | RE1-silencing transcription factor |
| RFX7 | regulatory factor X, 7 |
| RILPL2 | Rab interacting lysosomal protein-like 2 |
| RLIM | ring finger protein, LIM domain interacting |
| RNASE4 | ribonuclease, RNase A family, 4 |
| RNF115 | ring finger protein 115 |
| RNF168 | ring finger protein 168 |
| RNF185 | ring finger protein 185 |
| ROR1 | receptor tyrosine kinase-like orphan receptor 1 |
| RORA | RAR-related orphan receptor A |
| RPF2 | ribosome production factor 2 homolog (S. cerevisiae) |
| RPGR | retinitis pigmentosa GTPase regulator |
| RPH3AL | rabphilin 3A-like (without C2 domains) |
| RRP9 | ribosomal RNA processing 9, small subunit (SSU) processome component, homolog (yeast) |
| RSL24D1P3 | ribosomal L24 domain containing 1 pseudogene 3 |
| RWDD3 | RWD domain containing 3 |
| S100A16 | S100 calcium binding protein A16 |
| S100A6 | S100 calcium binding protein A6 |
| SAPS1 | SAPS domain family, member 1 |
| SC4MOL | sterol-C4-methyl oxidase-like |
| SDCCAG8 | serologically defined colon cancer antigen 8 |
| SDCCAG8 | serologically defined colon cancer antigen 8 |
| SEC62 | SEC62 homolog (S. cerevisiae) |
| SELT | selenoprotein T |
| SENP7 | SUMO1/sentrin specific peptidase 7 |
| SEPN1 | selenoprotein N, 1 |
| SEPP1 | selenoprotein P, plasma, 1 |
| SEPX1 | selenoprotein X, 1 |
| SERPINA3 | serpin peptidase inhibitor, clade A (alpha-1 antiproteinase, antitrypsin), member 3 |
| SESN1 | sestrin 1 |
| SFRS12 | splicing factor, arginine/serine-rich 12 |
| SFRS12IP1 | SFRS12-interacting protein 1 |
| SGK223 | homolog of rat pragma of Rnd2 |
| SH3BP5L | SH3-binding domain protein 5-like |
| SHROOM3 | shroom family member 3 |
| SHROOM3 | shroom family member 3 |
| SIRT5 | sirtuin (silent mating type information regulation 2 homolog) 5 (S. cerevisiae) |
| SIX5 | SIX homeobox 5 |
| SLC13A2 | solute carrier family 13 (sodium-dependent dicarboxylate transporter), member 2 |
| SLC16A5 | solute carrier family 16, member 5 (monocarboxylic acid transporter 6) |
| SLC20A1 | solute carrier family 20 (phosphate transporter), member 1 |
| SLC25A19 | solute carrier family 25 (mitochondrial thiamine pyrophosphate carrier), member 19 |
| SLC25A19 | solute carrier family 25 (mitochondrial thiamine pyrophosphate carrier), member 19 |
| SLC25A27 | solute carrier family 25, member 27 |
| SLC26A2 | solute carrier family 26 (sulfate transporter), member 2 |
| SLC27A4 | solute carrier family 27 (fatty acid transporter), member 4 |
| SLC38A11 | solute carrier family 38, member 11 |
| SLC38A2 | solute carrier family 38, member 2 |
| SLC38A4 | solute carrier family 38, member 4 |
| SLC45A3 | solute carrier family 45, member 3 |
| SLC7A11 | solute carrier family 7, (cationic amino acid transporter, y+ system) member 11 |
| SLC9A1 | solute carrier family 9 (sodium/hydrogen exchanger), member 1 |
| SMAD3 | SMAD family member 3 |
| SNAI2 | snail homolog 2 (Drosophila) |
| SPAG16 | sperm associated antigen 16 |
| SPHK1 | sphingosine kinase 1 |
| SPRY1 | sprouty homolog 1, antagonist of FGF signaling (Drosophila) |
| SQRDL | sulfide quinone reductase-like (yeast) |
| SRXN1 | sulfiredoxin 1 homolog (S. cerevisiae) |
| SSH2 | slingshot homolog 2 (Drosophila) |
| SSH3 | slingshot homolog 3 (Drosophila) |
| ST3GAL1 | ST3 beta-galactoside alpha-2,3-sialyltransferase 1 |
| STARD7 | StAR-related lipid transfer (START) domain containing 7 |
| STEAP2 | six transmembrane epithelial antigen of the prostate 2 |
| STK24 | serine/threonine kinase 24 (STE20 homolog, yeast) |
| STX1A | syntaxin 1A (brain) |
| SUV420H2 | suppressor of variegation 4-20 homolog 2 (Drosophila) |
| SVEP1 | sushi, von Willebrand factor type A, EGF and pentraxin domain containing 1 |
| SYTL1 | synaptotagmin-like 1 |
| TAF13 | TAF13 RNA polymerase II, TATA box binding protein (TBP)-associated factor, 18kDa |
| TAF1A | TATA box binding protein (TBP)-associated factor, RNA polymerase I, A, 48kDa |
| TAF1A | TATA box binding protein (TBP)-associated factor, RNA polymerase I, A, 48kDa |
| TAGLN | transgelin |
| TBC1D17 | TBC1 domain family, member 17 |
| TBX3 | T-box 3 |
| TCEA3 | transcription elongation factor A (SII), 3 |
| TCEAL6 | transcription elongation factor A (SII)-like 6 |
| TFPI | tissue factor pathway inhibitor (lipoprotein-associated coagulation inhibitor) |
| THBS2 | thrombospondin 2 |
| TLE1 | transducin-like enhancer of split 1 (E(sp1) homolog, Drosophila) |
| TLE4 | transducin-like enhancer of split 4 (E(sp1) homolog, Drosophila) |
| TLE4 | transducin-like enhancer of split 4 (E(sp1) homolog, Drosophila) |
| TMEM155 | transmembrane protein 155 |
| TMEM198 | transmembrane protein 198 |
| TMEM203 | transmembrane protein 203 |
| TMEM209 | transmembrane protein 209 |
| TMEM38B | transmembrane protein 38B |
| TMEM43 | transmembrane protein 43 |
| TMEM47 | transmembrane protein 47 |
| TNNC2 | troponin C type 2 (fast) |
| TOP1 | topoisomerase (DNA) I |
| TOX | thymocyte selection-associated high mobility group box |
| TP53 | tumor protein p53 |
| TP53 | tumor protein p53 |
| TP53INP2 | tumor protein p53 inducible nuclear protein 2 |
| TPD52L1 | tumor protein D52-like 1 |
| TRIM3 | tripartite motif-containing 3 |
| TRIO | triple functional domain (PTPRF interacting) |
| TRNP1 | TMF1-regulated nuclear protein 1 |
| TRPV2 | transient receptor potential cation channel, subfamily V, member 2 |
| TSIX | XIST antisense RNA (non-protein coding) |
| TSPAN2 | tetraspanin 2 |
| TTC26 | tetratricopeptide repeat domain 26 |
| TTC8 | tetratricopeptide repeat domain 8 |
| TTL | tubulin tyrosine ligase |
| TUB | tubby homolog (mouse) |
| TUBA1C | tubulin, alpha 1c |
| TUFT1 | tuftelin 1 |
| UBAP2L | ubiquitin associated protein 2-like |
| UBL4A | ubiquitin-like 4A |
| UBQLN4 | ubiquilin 4 |
| UBTD2 | ubiquitin domain containing 2 |
| UHRF2 | ubiquitin-like with PHD and ring finger domains 2 |
| UNK | unkempt homolog (Drosophila) |
| UNQ1870 | GALI1870 |
| UPF2 | UPF2 regulator of nonsense transcripts homolog (yeast) |
| USP12 | ubiquitin specific peptidase 12 |
| VCL | vinculin |
| VGLL3 | vestigial like 3 (Drosophila) |
| VPS18 | vacuolar protein sorting 18 homolog (S. cerevisiae) |
| WASF1 | WAS protein family, member 1 |
| WDR5 | WD repeat domain 5 |
| WDR89 | WD repeat domain 89 |
| WIPI1 | WD repeat domain, phosphoinositide interacting 1 |
| YAP1 | Yes-associated protein 1 |
| YPEL2 | yippee-like 2 (Drosophila) |
| YRDC | yrdC domain containing (E. coli) |
| YWHAG | tyrosine 3-monooxygenase/tryptophan 5-monooxygenase activation protein, gamma polypeptide |
| ZBTB22 | zinc finger and BTB domain containing 22 |
| ZBTB9 | zinc finger and BTB domain containing 9 |
| ZCCHC2 | zinc finger, CCHC domain containing 2 |
| ZCCHC5 | zinc finger, CCHC domain containing 5 |
| ZFP90 | zinc finger protein 90 homolog (mouse) |
| ZFP91 | zinc finger protein 91 homolog (mouse) |
| ZNF226 | zinc finger protein 226 |
| ZNF281 | zinc finger protein 281 |
| ZNF389 | zinc finger protein 389 |
| ZNF395 | zinc finger protein 395 |
| ZNF430 | zinc finger protein 430 |
| ZNF469 | zinc finger protein 469 |
| ZNF503 | zinc finger protein 503 |
| ZNF503 | zinc finger protein 503 |
| ZNF567 | zinc finger protein 567 |
| ZNF610 | zinc finger protein 610 |
| ZNF616 | zinc finger protein 616 |
| ZNF641 | zinc finger protein 641 |
| ZNF658 | zinc finger protein 658 |
| ZNF672 | zinc finger protein 672 |
| ZNF687 | zinc finger protein 687 |
| ZNF700 | zinc finger protein 700 |
| ZNF773 | zinc finger protein 773 |
| ZNF776 | zinc finger protein 776 |
| ZNF793 | zinc finger protein 793 |
| ***TCDD specific (n = 645)*** | |
| A1BG | alpha-1-B glycoprotein |
| ABCA5 | ATP-binding cassette, sub-family A (ABC1), member 5 |
| ABCB6 | ATP-binding cassette, sub-family B (MDR/TAP), member 6 |
| ABCC4 | ATP-binding cassette, sub-family C (CFTR/MRP), member 4 |
| ABTB1 | ankyrin repeat and BTB (POZ) domain containing 1 |
| ACOT1 | acyl-CoA thioesterase 1 |
| ADAL | adenosine deaminase-like |
| ADAMTS14 | ADAM metallopeptidase with thrombospondin type 1 motif, 14 |
| ADAT2 | adenosine deaminase, tRNA-specific 2, TAD2 homolog (S. cerevisiae) |
| ADCK4 | aarF domain containing kinase 4 |
| ADSS | adenylosuccinate synthase |
| AK5 | adenylate kinase 5 |
| ALDH16A1 | aldehyde dehydrogenase 16 family, member A1 |
| ALG13 | asparagine-linked glycosylation 13 homolog (S. cerevisiae) |
| ALKBH7 | alkB, alkylation repair homolog 7 (E. coli) |
| ANKZF1 | ankyrin repeat and zinc finger domain containing 1 |
| APITD1 | apoptosis-inducing, TAF9-like domain 1 |
| ARHGAP19 | Rho GTPase activating protein 19 |
| ARHGAP19 | Rho GTPase activating protein 19 |
| ARHGAP29 | Rho GTPase activating protein 29 |
| ARHGAP29 | Rho GTPase activating protein 29 |
| ARL6IP6 | ADP-ribosylation-like factor 6 interacting protein 6 |
| ARMC9 | armadillo repeat containing 9 |
| ARPP19 | cAMP-regulated phosphoprotein, 19kDa |
| ARSA | arylsulfatase A |
| ARSD | arylsulfatase D |
| ASCC2 | activating signal cointegrator 1 complex subunit 2 |
| ASF1B | ASF1 anti-silencing function 1 homolog B (S. cerevisiae) |
| ASPH | aspartate beta-hydroxylase |
| ATAD2 | ATPase family, AAA domain containing 2 |
| ATAD5 | ATPase family, AAA domain containing 5 |
| ATF4 | activating transcription factor 4 (tax-responsive enhancer element B67) |
| ATG4C | ATG4 autophagy related 4 homolog C (S. cerevisiae) |
| ATRIP | ATR interacting protein |
| ATXN7L1 | ataxin 7-like 1 |
| AURKB | aurora kinase B |
| B3GNTL1 | UDP-GlcNAc:betaGal beta-1,3-N-acetylglucosaminyltransferase-like 1 |
| BARD1 | BRCA1 associated RING domain 1 |
| BCAR3 | breast cancer anti-estrogen resistance 3 |
| BCAS4 | breast carcinoma amplified sequence 4 |
| BCL2L1 | BCL2-like 1 |
| BDP1 | B double prime 1, subunit of RNA polymerase III transcription initiation factor IIIB |
| BGN | biglycan |
| BMS1 | BMS1 homolog, ribosome assembly protein (yeast) |
| BRCA1 | breast cancer 1, early onset |
| BRCA2 | breast cancer 2, early onset |
| BRIP1 | BRCA1 interacting protein C-terminal helicase 1 |
| BTBD10 | BTB (POZ) domain containing 10 |
| BTNL2 | butyrophilin-like 2 (MHC class II associated) |
| BUD13 | BUD13 homolog (S. cerevisiae) |
| BZW1 | basic leucine zipper and W2 domains 1 |
| C10orf107 | chromosome 10 open reading frame 107 |
| C10orf119 | chromosome 10 open reading frame 119 |
| C12orf32 | chromosome 12 open reading frame 32 |
| C12orf48 | chromosome 12 open reading frame 48 |
| C14orf142 | chromosome 14 open reading frame 142 |
| C14orf179 | chromosome 14 open reading frame 179 |
| C14orf80 | chromosome 14 open reading frame 80 |
| C15orf42 | chromosome 15 open reading frame 42 |
| C16orf58 | chromosome 16 open reading frame 58 |
| C16orf59 | chromosome 16 open reading frame 59 |
| C17orf39 | chromosome 17 open reading frame 39 |
| C17orf76 | chromosome 17 open reading frame 76 |
| C18orf54 | chromosome 18 open reading frame 54 |
| C18orf55 | chromosome 18 open reading frame 55 |
| C18orf56 | chromosome 18 open reading frame 56 |
| C19orf48 | chromosome 19 open reading frame 48 |
| C19orf56 | chromosome 19 open reading frame 56 |
| C1orf54 | chromosome 1 open reading frame 54 |
| C1orf70 | chromosome 1 open reading frame 70 |
| C20orf132 | chromosome 20 open reading frame 132 |
| C20orf72 | chromosome 20 open reading frame 72 |
| C21orf58 | chromosome 21 open reading frame 58 |
| C21orf67 | chromosome 21 open reading frame 67 |
| C21orf93 | chromosome 21 open reading frame 93 |
| C22orf13 | chromosome 22 open reading frame 13 |
| C2orf56 | chromosome 2 open reading frame 56 |
| C3orf14 | chromosome 3 open reading frame 14 |
| C3orf18 | chromosome 3 open reading frame 18 |
| C4orf46 | chromosome 4 open reading frame 46 |
| C4orf46 | chromosome 4 open reading frame 46 |
| C5orf33 | chromosome 5 open reading frame 33 |
| C5orf37 | chromosome 5 open reading frame 37 |
| C5orf43 | chromosome 5 open reading frame 43 |
| C6orf103 | chromosome 6 open reading frame 103 |
| C8G | complement component 8, gamma polypeptide |
| C8orf48 | chromosome 8 open reading frame 48 |
| C9orf40 | chromosome 9 open reading frame 40 |
| C9orf40 | chromosome 9 open reading frame 40 |
| CAPN3 | calpain 3, (p94) |
| CAPZA1 | capping protein (actin filament) muscle Z-line, alpha 1 |
| CARD9 | caspase recruitment domain family, member 9 |
| CASP2 | caspase 2, apoptosis-related cysteine peptidase |
| CASP3 | caspase 3, apoptosis-related cysteine peptidase |
| CCDC109B | coiled-coil domain containing 109B |
| CCDC134 | coiled-coil domain containing 134 |
| CCDC82 | coiled-coil domain containing 82 |
| CCDC9 | coiled-coil domain containing 9 |
| CCNH | cyclin H |
| CCT8 | chaperonin containing TCP1, subunit 8 (theta) |
| CD59 | CD59 molecule, complement regulatory protein |
| CD79A | CD79a molecule, immunoglobulin-associated alpha |
| CD9 | CD9 molecule |
| CDC14B | CDC14 cell division cycle 14 homolog B (S. cerevisiae) |
| CDC45L | CDC45 cell division cycle 45-like (S. cerevisiae) |
| CDC7 | cell division cycle 7 homolog (S. cerevisiae) |
| CDCA5 | cell division cycle associated 5 |
| CDCA7 | cell division cycle associated 7 |
| CDKN2AIPNL | CDKN2A interacting protein N-terminal like |
| CENPI | centromere protein I |
| CENPJ | centromere protein J |
| CENPN | centromere protein N |
| CENPO | centromere protein O |
| CENPQ | centromere protein Q |
| CEP192 | centrosomal protein 192kDa |
| CES1 | carboxylesterase 1 (monocyte/macrophage serine esterase 1) |
| CES8 | carboxylesterase 8 (putative) |
| CFD | complement factor D (adipsin) |
| CHAF1B | chromatin assembly factor 1, subunit B (p60) |
| CHERP | calcium homeostasis endoplasmic reticulum protein |
| CIT | citron (rho-interacting, serine/threonine kinase 21) |
| CKAP2L | cytoskeleton associated protein 2-like |
| CKLF | chemokine-like factor |
| CLEC4M | C-type lectin domain family 4, member M |
| CNTLN | centlein, centrosomal protein |
| COL12A1 | collagen, type XII, alpha 1 |
| COL1A1 | collagen, type I, alpha 1 |
| COL27A1 | collagen, type XXVII, alpha 1 |
| COMMD6 | COMM domain containing 6 |
| COPS3 | COP9 constitutive photomorphogenic homolog subunit 3 (Arabidopsis) |
| COX11 | COX11 homolog, cytochrome c oxidase assembly protein (yeast) |
| CSE1L | CSE1 chromosome segregation 1-like (yeast) |
| CYB5R2 | cytochrome b5 reductase 2 |
| CYB5R2 | cytochrome b5 reductase 2 |
| DAGLB | diacylglycerol lipase, beta |
| DCAKD | dephospho-CoA kinase domain containing |
| DDX17 | DEAD (Asp-Glu-Ala-Asp) box polypeptide 17 |
| DDX23 | DEAD (Asp-Glu-Ala-Asp) box polypeptide 23 |
| DEM1 | defects in morphology 1 homolog (S. cerevisiae) |
| DENND3 | DENN/MADD domain containing 3 |
| DENND3 | DENN/MADD domain containing 3 |
| DEPDC1 | DEP domain containing 1 |
| DEPDC5 | DEP domain containing 5 |
| DERL3 | Der1-like domain family, member 3 |
| DGCR11 | DiGeorge syndrome critical region gene 11 |
| DKFZp686L14188 | hypothetical gene supported by BX538329 |
| DKFZp686O1327 | hypothetical gene supported by BC043549; BX648102 |
| DNA2 | DNA replication helicase 2 homolog (yeast) |
| DNAH11 | dynein, axonemal, heavy chain 11 |
| DNAJB2 | DnaJ (Hsp40) homolog, subfamily B, member 2 |
| DNAJC9 | DnaJ (Hsp40) homolog, subfamily C, member 9 |
| DONSON | downstream neighbor of SON |
| DPY19L1 | dpy-19-like 1 (C. elegans) |
| DSCC1 | defective in sister chromatid cohesion 1 homolog (S. cerevisiae) |
| DSN1 | DSN1, MIND kinetochore complex component, homolog (S. cerevisiae) |
| DTL | denticleless homolog (Drosophila) |
| DUS4L | dihydrouridine synthase 4-like (S. cerevisiae) |
| DUT | deoxyuridine triphosphatase |
| E2F2 | E2F transcription factor 2 |
| E2F7 | E2F transcription factor 7 |
| E2F8 | E2F transcription factor 8 |
| ECT2 | epithelial cell transforming sequence 2 oncogene |
| EEF1A1 | eukaryotic translation elongation factor 1 alpha 1 |
| EEF1A1 | eukaryotic translation elongation factor 1 alpha 1 |
| EEF1A1 | eukaryotic translation elongation factor 1 alpha 1 |
| EEF1A1P15 | eukaryotic translation elongation factor 1 alpha 1 pseudogene 15 |
| EID2B | EP300 interacting inhibitor of differentiation 2B |
| EIF1 | eukaryotic translation initiation factor 1 |
| EIF4A3 | eukaryotic translation initiation factor 4A3 |
| EME1 | essential meiotic endonuclease 1 homolog 1 (S. pombe) |
| EMP2 | epithelial membrane protein 2 |
| ENOPH1 | enolase-phosphatase 1 |
| ERBB2 | v-erb-b2 erythroblastic leukemia viral oncogene homolog 2, neuro/glioblastoma derived oncogene homolog (avian) |
| ERI1 | exoribonuclease 1 |
| ERI2 | ERI1 exoribonuclease family member 2 |
| ERMP1 | endoplasmic reticulum metallopeptidase 1 |
| ESPL1 | extra spindle pole bodies homolog 1 (S. cerevisiae) |
| ETS2 | v-ets erythroblastosis virus E26 oncogene homolog 2 (avian) |
| ETV1 | ets variant 1 |
| EXOSC8 | exosome component 8 |
| EZH2 | enhancer of zeste homolog 2 (Drosophila) |
| FABP4 | fatty acid binding protein 4, adipocyte |
| FADS3 | fatty acid desaturase 3 |
| FAF1 | Fas (TNFRSF6) associated factor 1 |
| FAM110A | family with sequence similarity 110, member A |
| FAM111A | family with sequence similarity 111, member A |
| FAM117A | family with sequence similarity 117, member A |
| FAM122B | family with sequence similarity 122B |
| FAM136A | family with sequence similarity 136, member A |
| FAM160A1 | family with sequence similarity 160, member A1 |
| FAM69A | family with sequence similarity 69, member A |
| FAM95B1 | family with sequence similarity 95, member B1 |
| FANCB | Fanconi anemia, complementation group B |
| FANCI | Fanconi anemia, complementation group I |
| FANCL | Fanconi anemia, complementation group L |
| FBXO2 | F-box protein 2 |
| FBXO41 | F-box protein 41 |
| FBXO43 | F-box protein 43 |
| FBXO5 | F-box protein 5 |
| FEN1 | flap structure-specific endonuclease 1 |
| FKBP5 | FK506 binding protein 5 |
| FLJ33630 | hypothetical LOC644873 |
| FLJ38717 | FLJ38717 protein |
| FLNA | filamin A, alpha |
| FOLR3 | folate receptor 3 (gamma) |
| FOXD1 | forkhead box D1 |
| FOXRED1 | FAD-dependent oxidoreductase domain containing 1 |
| FSCN2 | fascin homolog 2, actin-bundling protein, retinal (Strongylocentrotus purpuratus) |
| FTCD | formiminotransferase cyclodeaminase |
| FTSJ1 | FtsJ homolog 1 (E. coli) |
| GGT7 | gamma-glutamyltransferase 7 |
| GLMN | glomulin, FKBP associated protein |
| GLO1 | glyoxalase I |
| GLTSCR1 | glioma tumor suppressor candidate region gene 1 |
| GLTSCR2 | glioma tumor suppressor candidate region gene 2 |
| GLYATL1 | glycine-N-acyltransferase-like 1 |
| GMNN | geminin, DNA replication inhibitor |
| GNPTAB | N-acetylglucosamine-1-phosphate transferase, alpha and beta subunits |
| GOLGA6L9 | golgin A6 family-like 9 |
| GOLGA8A | golgin A8 family, member A |
| GOLGA9P | golgin A9 (pseudogene) |
| GPN1 | GPN-loop GTPase 1 |
| GPR135 | G protein-coupled receptor 135 |
| H19 | H19, imprinted maternally expressed transcript (non-protein coding) |
| H3F3B | H3 histone, family 3B (H3.3B) |
| HAUS5 | HAUS augmin-like complex, subunit 5 |
| HAUS5 | HAUS augmin-like complex, subunit 5 |
| HAUS8 | HAUS augmin-like complex, subunit 8 |
| HBG1 | hemoglobin, gamma A |
| hCG_1820661 | hypothetical LOC400752 |
| HELZ | helicase with zinc finger |
| HERPUD1 | homocysteine-inducible, endoplasmic reticulum stress-inducible, ubiquitin-like domain member 1 |
| HIPK2 | homeodomain interacting protein kinase 2 |
| HIRIP3 | HIRA interacting protein 3 |
| HIST1H1B | histone cluster 1, H1b |
| HIST1H1D | histone cluster 1, H1d |
| HIST1H2AA | histone cluster 1, H2aa |
| HIST1H2AI | histone cluster 1, H2ai |
| HIST1H3B | histone cluster 1, H3b |
| HIST1H3F | histone cluster 1, H3f |
| HIST1H3H | histone cluster 1, H3h |
| HIST1H3J | histone cluster 1, H3j |
| HIST1H4D | histone cluster 1, H4d |
| HIST1H4L | histone cluster 1, H4l |
| HIST2H2AB | histone cluster 2, H2ab |
| HIST2H2BE | histone cluster 2, H2be |
| HJURP | Holliday junction recognition protein |
| HLA-A | major histocompatibility complex, class I, A |
| HLA-G | major histocompatibility complex, class I, G |
| HLTF | helicase-like transcription factor |
| HMGB3 | high-mobility group box 3 |
| HMMR | hyaluronan-mediated motility receptor (RHAMM) |
| HNRNPC | heterogeneous nuclear ribonucleoprotein C (C1/C2) |
| HNRNPF | heterogeneous nuclear ribonucleoprotein F |
| HNRPDL | heterogeneous nuclear ribonucleoprotein D-like |
| HOTAIR | hox transcript antisense RNA (non-protein coding) |
| HPRT1 | hypoxanthine phosphoribosyltransferase 1 |
| HS2ST1 | heparan sulfate 2-O-sulfotransferase 1 |
| HSPA9 | heat shock 70kDa protein 9 (mortalin) |
| HVCN1 | hydrogen voltage-gated channel 1 |
| ICAM4 | intercellular adhesion molecule 4 (Landsteiner-Wiener blood group) |
| ICMT | isoprenylcysteine carboxyl methyltransferase |
| IFITM2 | interferon induced transmembrane protein 2 (1-8D) |
| IFITM3 | interferon induced transmembrane protein 3 (1-8U) |
| IGBP1 | immunoglobulin (CD79A) binding protein 1 |
| IGLV2-14 | immunoglobulin lambda variable 2-14 |
| INO80 | INO80 homolog (S. cerevisiae) |
| INTS5 | integrator complex subunit 5 |
| INTS7 | integrator complex subunit 7 |
| INTU | inturned planar cell polarity effector homolog (Drosophila) |
| IP6K2 | inositol hexakisphosphate kinase 2 |
| IPO11 | importin 11 |
| ITGB3BP | integrin beta 3 binding protein (beta3-endonexin) |
| JDP2 | Jun dimerization protein 2 |
| KCTD9 | potassium channel tetramerisation domain containing 9 |
| KDM4A | lysine (K)-specific demethylase 4A |
| KDM4B | lysine (K)-specific demethylase 4B |
| KDM4D | lysine (K)-specific demethylase 4D |
| KHDRBS3 | KH domain containing, RNA binding, signal transduction associated 3 |
| KIAA0495 | KIAA0495 |
| KIAA0913 | KIAA0913 |
| KIAA1279 | KIAA1279 |
| KIAA1841 | KIAA1841 |
| KIF14 | kinesin family member 14 |
| KLF7 | Kruppel-like factor 7 (ubiquitous) |
| KLHL28 | kelch-like 28 (Drosophila) |
| KLHL35 | kelch-like 35 (Drosophila) |
| LAMB2 | laminin, beta 2 (laminin S) |
| LAMB2 | laminin, beta 2 (laminin S) |
| LAMC2 | laminin, gamma 2 |
| LAYN | layilin |
| LCMT2 | leucine carboxyl methyltransferase 2 |
| LDHA | lactate dehydrogenase A |
| LIN52 | lin-52 homolog (C. elegans) |
| LIN9 | lin-9 homolog (C. elegans) |
| LOC100128028 | hypothetical protein LOC100128028 |
| LOC100128191 | hypothetical protein LOC100128191 |
| LOC100128469 | small nuclear ribonucleoprotein polypeptide C pseudogene |
| LOC100128567 | hypothetical protein LOC100128567 |
| LOC100129387 | hypothetical LOC100129387 |
| LOC100129596 | hypothetical protein LOC100129596 |
| LOC100130654 | hypothetical protein LOC100130654 |
| LOC100130710 | hypothetical LOC100130710 |
| LOC100132015 | similar to testis expressed sequence 13A |
| LOC100132273 | hypothetical protein LOC100132273 |
| LOC100133554 | hypothetical protein LOC100133554 |
| LOC100190939 | hypothetical LOC100190939 |
| LOC144571 | hypothetical protein LOC144571 |
| LOC145216 | hypothetical LOC145216 |
| LOC149351 | hypothetical protein LOC149351 |
| LOC222159 | hypothetical protein LOC222159 |
| LOC344382 | similar to unr-interacting protein |
| LOC349114 | hypothetical LOC349114 |
| LOC389493 | hypothetical protein LOC389493 |
| LOC399804 | similar to nucleophosmin 1 |
| LOC400236 | hypothetical LOC400236 |
| LOC402360 | similar to hCG1742476 |
| LOC440900 | hypothetical LOC440900 |
| LOC441644 | REST corepressor 2 pseudogene |
| LOC647979 | hypothetical LOC647979 |
| LOC727788 | hypothetical LOC727788 |
| LOC730144 | similar to eukaryotic translation initiation factor 1 |
| LOC81691 | exonuclease NEF-sp |
| LOC84931 | hypothetical LOC84931 |
| LOC91316 | glucuronidase, beta/immunoglobulin lambda-like polypeptide 1 pseudogene |
| LRRC16B | leucine rich repeat containing 16B |
| LTA4H | leukotriene A4 hydrolase |
| LYPLA1 | lysophospholipase I |
| LYRM5 | LYR motif containing 5 |
| LYRM7 | Lyrm7 homolog (mouse) |
| LYST | lysosomal trafficking regulator |
| MANEA | mannosidase, endo-alpha |
| MAP2K6 | mitogen-activated protein kinase kinase 6 |
| MAP3K5 | mitogen-activated protein kinase kinase kinase 5 |
| MAPRE1 | microtubule-associated protein, RP/EB family, member 1 |
| MARK4 | MAP/microtubule affinity-regulating kinase 4 |
| MBL1P | mannose-binding lectin (protein A) 1, pseudogene |
| MCM2 | minichromosome maintenance complex component 2 |
| MCM3 | minichromosome maintenance complex component 3 |
| MCM5 | minichromosome maintenance complex component 5 |
| MCM7 | minichromosome maintenance complex component 7 |
| MCM8 | minichromosome maintenance complex component 8 |
| MELK | maternal embryonic leucine zipper kinase |
| METAP1 | methionyl aminopeptidase 1 |
| MGC23284 | hypothetical LOC197187 |
| MINPP1 | multiple inositol polyphosphate histidine phosphatase, 1 |
| MIS12 | MIS12, MIND kinetochore complex component, homolog (S. pombe) |
| MLLT3 | myeloid/lymphoid or mixed-lineage leukemia (trithorax homolog, Drosophila); translocated to, 3 |
| MMP19 | matrix metallopeptidase 19 |
| MMP19 | matrix metallopeptidase 19 |
| MMP2 | matrix metallopeptidase 2 (gelatinase A, 72kDa gelatinase, 72kDa type IV collagenase) |
| MND1 | meiotic nuclear divisions 1 homolog (S. cerevisiae) |
| MOSPD1 | motile sperm domain containing 1 |
| MPDU1 | mannose-P-dolichol utilization defect 1 |
| MPHOSPH8 | M-phase phosphoprotein 8 |
| MRC2 | mannose receptor, C type 2 |
| MRPL17 | mitochondrial ribosomal protein L17 |
| MRPL39 | mitochondrial ribosomal protein L39 |
| MTBP | Mdm2, transformed 3T3 cell double minute 2, p53 binding protein (mouse) binding protein, 104kDa |
| MTERFD1 | MTERF domain containing 1 |
| MTMR12 | myotubularin related protein 12 |
| MTMR6 | myotubularin related protein 6 |
| MTRF1L | mitochondrial translational release factor 1-like |
| MTSS1L | metastasis suppressor 1-like |
| MUC2 | mucin 2, oligomeric mucus/gel-forming |
| MXRA8 | matrix-remodelling associated 8 |
| MYRIP | myosin VIIA and Rab interacting protein |
| MYST2 | MYST histone acetyltransferase 2 |
| NAP1L4 | nucleosome assembly protein 1-like 4 |
| NASP | nuclear autoantigenic sperm protein (histone-binding) |
| NAV2 | neuron navigator 2 |
| NCAPD3 | non-SMC condensin II complex, subunit D3 |
| NCAPH2 | non-SMC condensin II complex, subunit H2 |
| NCKAP5L | NCK-associated protein 5-like |
| NCOA5 | nuclear receptor coactivator 5 |
| NCRNA00107 | non-protein coding RNA 107 |
| NDC80 | NDC80 homolog, kinetochore complex component (S. cerevisiae) |
| NDRG3 | NDRG family member 3 |
| NDST1 | N-deacetylase/N-sulfotransferase (heparan glucosaminyl) 1 |
| NEIL3 | nei endonuclease VIII-like 3 (E. coli) |
| NFE2L3 | nuclear factor (erythroid-derived 2)-like 3 |
| NFKBIL2 | nuclear factor of kappa light polypeptide gene enhancer in B-cells inhibitor-like 2 |
| NHS | Nance-Horan syndrome (congenital cataracts and dental anomalies) |
| NMNAT1 | nicotinamide nucleotide adenylyltransferase 1 |
| NPAS2 | neuronal PAS domain protein 2 |
| NRIP1 | nuclear receptor interacting protein 1 |
| NRM | nurim (nuclear envelope membrane protein) |
| NSMCE4A | non-SMC element 4 homolog A (S. cerevisiae) |
| NT5E | 5'-nucleotidase, ecto (CD73) |
| NTM | neurotrimin |
| NUDT17 | nudix (nucleoside diphosphate linked moiety X)-type motif 17 |
| NUDT21 | nudix (nucleoside diphosphate linked moiety X)-type motif 21 |
| NUP205 | nucleoporin 205kDa |
| NUP35 | nucleoporin 35kDa |
| NUP50 | nucleoporin 50kDa |
| NUPL1 | nucleoporin like 1 |
| OGFRL1 | opioid growth factor receptor-like 1 |
| OLA1 | Obg-like ATPase 1 |
| OR5A2 | olfactory receptor, family 5, subfamily A, member 2 |
| ORC1L | origin recognition complex, subunit 1-like (yeast) |
| ORC5L | origin recognition complex, subunit 5-like (yeast) |
| ORC6L | origin recognition complex, subunit 6 like (yeast) |
| OSBPL1A | oxysterol binding protein-like 1A |
| OSBPL8 | oxysterol binding protein-like 8 |
| OXER1 | oxoeicosanoid (OXE) receptor 1 |
| P2RX4 | purinergic receptor P2X, ligand-gated ion channel, 4 |
| PAFAH1B2 | platelet-activating factor acetylhydrolase 1b, catalytic subunit 2 (30kDa) |
| PAFAH1B3 | platelet-activating factor acetylhydrolase 1b, catalytic subunit 3 (29kDa) |
| PALB2 | partner and localizer of BRCA2 |
| PANK4 | pantothenate kinase 4 |
| PARP1 | poly (ADP-ribose) polymerase 1 |
| PARP2 | poly (ADP-ribose) polymerase 2 |
| PCID2 | PCI domain containing 2 |
| PCMTD2 | protein-L-isoaspartate (D-aspartate) O-methyltransferase domain containing 2 |
| PCNA | proliferating cell nuclear antigen |
| PCNA | proliferating cell nuclear antigen |
| PDLIM7 | PDZ and LIM domain 7 (enigma) |
| PEX3 | peroxisomal biogenesis factor 3 |
| PHB | prohibitin |
| PHF15 | PHD finger protein 15 |
| PHF19 | PHD finger protein 19 |
| PHF20L1 | PHD finger protein 20-like 1 |
| PHF20L1 | PHD finger protein 20-like 1 |
| PI4K2B | phosphatidylinositol 4-kinase type 2 beta |
| PICK1 | protein interacting with PRKCA 1 |
| PLCB4 | phospholipase C, beta 4 |
| PLD2 | phospholipase D2 |
| PLEKHH3 | pleckstrin homology domain containing, family H (with MyTH4 domain) member 3 |
| PLP2 | proteolipid protein 2 (colonic epithelium-enriched) |
| PMS2L11 | postmeiotic segregation increased 2-like 11 pseudogene |
| POLD1 | polymerase (DNA directed), delta 1, catalytic subunit 125kDa |
| POLD3 | polymerase (DNA-directed), delta 3, accessory subunit |
| POLE | polymerase (DNA directed), epsilon |
| POLE3 | polymerase (DNA directed), epsilon 3 (p17 subunit) |
| POT1 | POT1 protection of telomeres 1 homolog (S. pombe) |
| PPP1CC | protein phosphatase 1, catalytic subunit, gamma isozyme |
| PPP1R8 | protein phosphatase 1, regulatory (inhibitor) subunit 8 |
| PRMT3 | protein arginine methyltransferase 3 |
| PRMT7 | protein arginine methyltransferase 7 |
| PRO2852 | hypothetical protein PRO2852 |
| PRPF40B | PRP40 pre-mRNA processing factor 40 homolog B (S. cerevisiae) |
| PSMB2 | proteasome (prosome, macropain) subunit, beta type, 2 |
| PSMC3IP | PSMC3 interacting protein |
| PSMD2 | proteasome (prosome, macropain) 26S subunit, non-ATPase, 2 |
| PTH2 | parathyroid hormone 2 |
| PTPDC1 | protein tyrosine phosphatase domain containing 1 |
| PTPLAD1 | protein tyrosine phosphatase-like A domain containing 1 |
| PXK | PX domain containing serine/threonine kinase |
| RAB30 | RAB30, member RAS oncogene family |
| RAB3B | RAB3B, member RAS oncogene family |
| RAB9B | RAB9B, member RAS oncogene family |
| RAD51L1 | RAD51-like 1 (S. cerevisiae) |
| RAD51L3 | RAD51-like 3 (S. cerevisiae) |
| RAD54L | RAD54-like (S. cerevisiae) |
| RANBP6 | RAN binding protein 6 |
| RAPGEF6 | Rap guanine nucleotide exchange factor (GEF) 6 |
| RASSF7 | Ras association (RalGDS/AF-6) domain family (N-terminal) member 7 |
| RBBP4 | retinoblastoma binding protein 4 |
| RBBP8 | retinoblastoma binding protein 8 |
| RBCK1 | RanBP-type and C3HC4-type zinc finger containing 1 |
| RDX | radixin |
| REL | v-rel reticuloendotheliosis viral oncogene homolog (avian) |
| REV3L | REV3-like, catalytic subunit of DNA polymerase zeta (yeast) |
| RFC2 | replication factor C (activator 1) 2, 40kDa |
| RFC4 | replication factor C (activator 1) 4, 37kDa |
| RGMA | RGM domain family, member A |
| RGNEF | Rho-guanine nucleotide exchange factor |
| RHOQ | ras homolog gene family, member Q |
| RNASEH2B | ribonuclease H2, subunit B |
| RNF19B | ring finger protein 19B |
| RNF4 | ring finger protein 4 |
| RNPC3 | RNA-binding region (RNP1, RRM) containing 3 |
| RNPS1 | RNA binding protein S1, serine-rich domain |
| RPS27 | ribosomal protein S27 |
| RQCD1 | RCD1 required for cell differentiation1 homolog (S. pombe) |
| RRM2 | ribonucleotide reductase M2 |
| RRP15 | ribosomal RNA processing 15 homolog (S. cerevisiae) |
| RSL24D1P11 | ribosomal L24 domain containing 1 pseudogene 11 |
| SAMD14 | sterile alpha motif domain containing 14 |
| SAMD4B | sterile alpha motif domain containing 4B |
| SAT2 | spermidine/spermine N1-acetyltransferase family member 2 |
| SATB1 | SATB homeobox 1 |
| SCAND2 | SCAN domain containing 2 pseudogene |
| SCAND3 | SCAN domain containing 3 |
| SCARNA2 | small Cajal body-specific RNA 2 |
| SCML2 | sex comb on midleg-like 2 (Drosophila) |
| SENP1 | SUMO1/sentrin specific peptidase 1 |
| SENP2 | SUMO1/sentrin/SMT3 specific peptidase 2 |
| SERHL2 | serine hydrolase-like 2 |
| SERPINE1 | serpin peptidase inhibitor, clade E (nexin, plasminogen activator inhibitor type 1), member 1 |
| SEZ6 | seizure related 6 homolog (mouse) |
| SFMBT1 | Scm-like with four mbt domains 1 |
| SGOL2 | shugoshin-like 2 (S. pombe) |
| SHISA4 | shisa homolog 4 (Xenopus laevis) |
| SHISA5 | shisa homolog 5 (Xenopus laevis) |
| SHMT1 | serine hydroxymethyltransferase 1 (soluble) |
| SHMT1 | serine hydroxymethyltransferase 1 (soluble) |
| SIRPA | signal-regulatory protein alpha |
| SIRPB2 | signal-regulatory protein beta 2 |
| SKA3 | spindle and kinetochore associated complex subunit 3 |
| SLBP | stem-loop binding protein |
| SLC13A3 | solute carrier family 13 (sodium-dependent dicarboxylate transporter), member 3 |
| SLC22A5 | solute carrier family 22 (organic cation/carnitine transporter), member 5 |
| SLC23A3 | solute carrier family 23 (nucleobase transporters), member 3 |
| SLC25A45 | solute carrier family 25, member 45 |
| SLC27A1 | solute carrier family 27 (fatty acid transporter), member 1 |
| SLC35E3 | solute carrier family 35, member E3 |
| SLC40A1 | solute carrier family 40 (iron-regulated transporter), member 1 |
| SLC46A1 | solute carrier family 46 (folate transporter), member 1 |
| SLC5A2 | solute carrier family 5 (sodium/glucose cotransporter), member 2 |
| SLC7A1 | solute carrier family 7 (cationic amino acid transporter, y+ system), member 1 |
| SLC7A7 | solute carrier family 7 (cationic amino acid transporter, y+ system), member 7 |
| SLK | STE20-like kinase (yeast) |
| SMARCD3 | SWI/SNF related, matrix associated, actin dependent regulator of chromatin, subfamily d, member 3 |
| SMC3 | structural maintenance of chromosomes 3 |
| SNORD15A | small nucleolar RNA, C/D box 15A |
| SNRNP27 | small nuclear ribonucleoprotein 27kDa (U4/U6.U5) |
| SPEG | SPEG complex locus |
| SRFBP1 | serum response factor binding protein 1 |
| SRR | serine racemase |
| SSFA2 | sperm specific antigen 2 |
| SSTR3 | somatostatin receptor 3 |
| STIL | SCL/TAL1 interrupting locus |
| STK24 | serine/threonine kinase 24 (STE20 homolog, yeast) |
| STK36 | serine/threonine kinase 36, fused homolog (Drosophila) |
| STX16 | syntaxin 16 |
| SUB1 | SUB1 homolog (S. cerevisiae) |
| SUMF1 | sulfatase modifying factor 1 |
| SYNJ2 | synaptojanin 2 |
| TBC1D2 | TBC1 domain family, member 2 |
| TEAD2 | TEA domain family member 2 |
| TEAD3 | TEA domain family member 3 |
| TEP1 | telomerase-associated protein 1 |
| TET2 | tet oncogene family member 2 |
| TFAM | transcription factor A, mitochondrial |
| TFAM | transcription factor A, mitochondrial |
| TFDP1 | transcription factor Dp-1 |
| TFDP1 | transcription factor Dp-1 |
| TGFB1I1 | transforming growth factor beta 1 induced transcript 1 |
| TGFBRAP1 | transforming growth factor, beta receptor associated protein 1 |
| THOC1 | THO complex 1 |
| TLE1 | transducin-like enhancer of split 1 (E(sp1) homolog, Drosophila) |
| TM4SF1 | transmembrane 4 L six family member 1 |
| TM4SF1 | transmembrane 4 L six family member 1 |
| TMEM161A | transmembrane protein 161A |
| TMEM209 | transmembrane protein 209 |
| TMEM55A | transmembrane protein 55A |
| TMEM95 | transmembrane protein 95 |
| TMEM97 | transmembrane protein 97 |
| TNAP | TRAFs and NIK-associated protein |
| TNPO1 | transportin 1 |
| TNPO3 | transportin 3 |
| TNRC18 | trinucleotide repeat containing 18 |
| TNRC18 | trinucleotide repeat containing 18 |
| TNRC6C | trinucleotide repeat containing 6C |
| TPM2 | tropomyosin 2 (beta) |
| TPM2 | tropomyosin 2 (beta) |
| TPM3 | tropomyosin 3 |
| TRDMT1 | tRNA aspartic acid methyltransferase 1 |
| TRIM16L | tripartite motif-containing 16-like |
| TRIM26 | tripartite motif-containing 26 |
| TRIP13 | thyroid hormone receptor interactor 13 |
| TRMT2B | TRM2 tRNA methyltransferase 2 homolog B (S. cerevisiae) |
| TSFM | Ts translation elongation factor, mitochondrial |
| TTC39B | tetratricopeptide repeat domain 39B |
| TTF2 | transcription termination factor, RNA polymerase II |
| TTK | TTK protein kinase |
| TUBGCP3 | tubulin, gamma complex associated protein 3 |
| TUBGCP5 | tubulin, gamma complex associated protein 5 |
| U2AF1L4 | U2 small nuclear RNA auxiliary factor 1-like 4 |
| UAP1 | UDP-N-acteylglucosamine pyrophosphorylase 1 |
| UBA2 | ubiquitin-like modifier activating enzyme 2 |
| UBA6 | ubiquitin-like modifier activating enzyme 6 |
| UBE2E3 | ubiquitin-conjugating enzyme E2E 3 (UBC4/5 homolog, yeast) |
| UBE2J2 | ubiquitin-conjugating enzyme E2, J2 (UBC6 homolog, yeast) |
| UBE2T | ubiquitin-conjugating enzyme E2T (putative) |
| UBE4B | ubiquitination factor E4B (UFD2 homolog, yeast) |
| UBN2 | ubinuclein 2 |
| UBQLNL | ubiquilin-like |
| UBR7 | ubiquitin protein ligase E3 component n-recognin 7 (putative) |
| UCP3 | uncoupling protein 3 (mitochondrial, proton carrier) |
| ULK1 | unc-51-like kinase 1 (C. elegans) |
| UNC119B | unc-119 homolog B (C. elegans) |
| UNQ2963 | hypothetical LOC283314 |
| USF2 | upstream transcription factor 2, c-fos interacting |
| USP10 | ubiquitin specific peptidase 10 |
| USP39 | ubiquitin specific peptidase 39 |
| USP53 | ubiquitin specific peptidase 53 |
| USP53 | ubiquitin specific peptidase 53 |
| VAMP2 | vesicle-associated membrane protein 2 (synaptobrevin 2) |
| VMA21 | VMA21 vacuolar H+-ATPase homolog (S. cerevisiae) |
| VOPP1 | vesicular, overexpressed in cancer, prosurvival protein 1 |
| VPRBP | Vpr (HIV-1) binding protein |
| VPS13B | vacuolar protein sorting 13 homolog B (yeast) |
| VRK1 | vaccinia related kinase 1 |
| WASH1 | WAS protein family homolog 1 |
| WDHD1 | WD repeat and HMG-box DNA binding protein 1 |
| WDR37 | WD repeat domain 37 |
| WDR76 | WD repeat domain 76 |
| WDR90 | WD repeat domain 90 |
| WRAP53 | WD repeat containing, antisense to TP53 |
| XPA | xeroderma pigmentosum, complementation group A |
| XPO4 | exportin 4 |
| YEATS4 | YEATS domain containing 4 |
| YTHDF1 | YTH domain family, member 1 |
| YTHDF2 | YTH domain family, member 2 |
| ZBED4 | zinc finger, BED-type containing 4 |
| ZFP91 | zinc finger protein 91 homolog (mouse) |
| ZMYM2 | zinc finger, MYM-type 2 |
| ZMYND8 | zinc finger, MYND-type containing 8 |
| ZNF100 | zinc finger protein 100 |
| ZNF132 | zinc finger protein 132 |
| ZNF180 | zinc finger protein 180 |
| ZNF184 | zinc finger protein 184 |
| ZNF197 | zinc finger protein 197 |
| ZNF234 | zinc finger protein 234 |
| ZNF264 | zinc finger protein 264 |
| ZNF33A | zinc finger protein 33A |
| ZNF33A | zinc finger protein 33A |
| ZNF367 | zinc finger protein 367 |
| ZNF367 | zinc finger protein 367 |
| ZNF37B | zinc finger protein 37B (pseudogene) |
| ZNF460 | zinc finger protein 460 |
| ZNF550 | zinc finger protein 550 |
| ZNF551 | zinc finger protein 551 |
| ZNF555 | zinc finger protein 555 |
| ZNF561 | zinc finger protein 561 |
| ZNF606 | zinc finger protein 606 |
| ZNF630 | zinc finger protein 630 |
| ZNF641 | zinc finger protein 641 |
| ZNF677 | zinc finger protein 677 |
| ZNF684 | zinc finger protein 684 |
| ZNF684 | zinc finger protein 684 |
| ZNF70 | zinc finger protein 70 |
| ZNF714 | zinc finger protein 714 |
| ZNF728 | zinc finger protein 728 |
| ZNF730 | zinc finger protein 730 |
| ZNF788 | zinc finger family member 788 |
| ZNF799 | zinc finger protein 799 |
| ZNF800 | zinc finger protein 800 |
| ZNF846 | zinc finger protein 846 |
| ZNF879 | zinc finger protein 879 |
| ZNF91 | zinc finger protein 91 |
| ZNF93 | zinc finger protein 93 |
| ZWINT | ZW10 interactor |
| ***BPA-E2 common (n = 17)*** | |
| C1orf203 | chromosome 1 open reading frame 203 |
| CCDC134 | coiled-coil domain containing 134 |
| CCDC41 | coiled-coil domain containing 41 |
| DCUN1D2 | DCN1, defective in cullin neddylation 1, domain containing 2 (S. cerevisiae) |
| EXOC8 | exocyst complex component 8 |
| HEATR3 | HEAT repeat containing 3 |
| LOC645967 | hypothetical LOC645967 |
| LRCH1 | leucine-rich repeats and calponin homology (CH) domain containing 1 |
| MMP11 | matrix metallopeptidase 11 (stromelysin 3) |
| MYST3 | MYST histone acetyltransferase (monocytic leukemia) 3 |
| NHLRC2 | NHL repeat containing 2 |
| TBC1D22B | TBC1 domain family, member 22B |
| TMEM8B | transmembrane protein 8B |
| ZNF224 | zinc finger protein 224 |
| ZNF429 | zinc finger protein 429 |
| ZNF461 | zinc finger protein 461 |
| ZNF546 | zinc finger protein 546 |
| ***BPA-TCDD common (n = 10)*** | |
| CRTC1 | CREB regulated transcription coactivator 1 |
| HS2ST1 | heparan sulfate 2-O-sulfotransferase 1 |
| JRKL | jerky homolog-like (mouse) |
| LDLRAD3 | low density lipoprotein receptor class A domain containing 3 |
| MCM9 | minichromosome maintenance complex component 9 |
| NBEAL1 | neurobeachin-like 1 |
| NEK10 | NIMA (never in mitosis gene a)- related kinase 10 |
| NFRKB | nuclear factor related to kappaB binding protein |
| PAEP | progestagen-associated endometrial protein |
| USP11 | ubiquitin specific peptidase 11 |
| ***E2-TCDD common (n = 168)*** | |
| ABL2 | v-abl Abelson murine leukemia viral oncogene homolog 2 (arg, Abelson-related gene) |
| ACSF2 | acyl-CoA synthetase family member 2 |
| ACTA2 | actin, alpha 2, smooth muscle, aorta |
| AGBL5 | ATP/GTP binding protein-like 5 |
| AHRR | aryl-hydrocarbon receptor repressor |
| AMOTL2 | angiomotin like 2 |
| ANKRD9 | ankyrin repeat domain 9 |
| ANO7 | anoctamin 7 |
| AP1G2 | adaptor-related protein complex 1, gamma 2 subunit |
| APBB3 | amyloid beta (A4) precursor protein-binding, family B, member 3 |
| APOL4 | apolipoprotein L, 4 |
| ASAP1 | ArfGAP with SH3 domain, ankyrin repeat and PH domain 1 |
| ASB2 | ankyrin repeat and SOCS box-containing 2 |
| ASS1 | argininosuccinate synthetase 1 |
| ASS1 | argininosuccinate synthetase 1 |
| ATXN1 | ataxin 1 |
| BCAP29 | B-cell receptor-associated protein 29 |
| BZW1 | basic leucine zipper and W2 domains 1 |
| C1orf177 | chromosome 1 open reading frame 177 |
| C2orf69 | chromosome 2 open reading frame 69 |
| C3orf26 | chromosome 3 open reading frame 26 |
| C8orf4 | chromosome 8 open reading frame 4 |
| C9orf167 | chromosome 9 open reading frame 167 |
| C9orf24 | chromosome 9 open reading frame 24 |
| C9orf7 | chromosome 9 open reading frame 7 |
| C9orf9 | chromosome 9 open reading frame 9 |
| CALD1 | caldesmon 1 |
| CCDC43 | coiled-coil domain containing 43 |
| CCNE1 | cyclin E1 |
| CD44 | CD44 molecule (Indian blood group) |
| CDT1 | chromatin licensing and DNA replication factor 1 |
| CHAF1A | chromatin assembly factor 1, subunit A (p150) |
| CISD1 | CDGSH iron sulfur domain 1 |
| CNN2 | calponin 2 |
| COL17A1 | collagen, type XVII, alpha 1 |
| COL1A2 | collagen, type I, alpha 2 |
| CPAMD8 | C3 and PZP-like, alpha-2-macroglobulin domain containing 8 |
| CRYAB | crystallin, alpha B |
| CSDC2 | cold shock domain containing C2, RNA binding |
| DAAM2 | dishevelled associated activator of morphogenesis 2 |
| DCN | decorin |
| DDX11 | DEAD/H (Asp-Glu-Ala-Asp/His) box polypeptide 11 (CHL1-like helicase homolog, S. cerevisiae) |
| DEPDC6 | DEP domain containing 6 |
| DLEU1 | deleted in lymphocytic leukemia 1 (non-protein coding) |
| DMPK | dystrophia myotonica-protein kinase |
| E2F7 | E2F transcription factor 7 |
| ECHDC2 | enoyl Coenzyme A hydratase domain containing 2 |
| EFTUD1 | elongation factor Tu GTP binding domain containing 1 |
| EIF1AX | eukaryotic translation initiation factor 1A, X-linked |
| EIF1AX | eukaryotic translation initiation factor 1A, X-linked |
| EIF1AX | eukaryotic translation initiation factor 1A, X-linked |
| EPHB1 | EPH receptor B1 |
| ETV4 | ets variant 4 |
| EXTL1 | exostoses (multiple)-like 1 |
| FAM107B | family with sequence similarity 107, member B |
| FAM123B | family with sequence similarity 123B |
| FAM129B | family with sequence similarity 129, member B |
| FAM180A | family with sequence similarity 180, member A |
| FAM66A | family with sequence similarity 66, member A |
| FANCG | Fanconi anemia, complementation group G |
| FBLN5 | fibulin 5 |
| FCRLB | Fc receptor-like B |
| FER1L4 | fer-1-like 4 (C. elegans) |
| FHL1 | four and a half LIM domains 1 |
| FTL | ferritin, light polypeptide |
| FZD4 | frizzled homolog 4 (Drosophila) |
| GK | glycerol kinase |
| GLIS1 | GLIS family zinc finger 1 |
| GOPC | golgi-associated PDZ and coiled-coil motif containing |
| GSTO1 | glutathione S-transferase omega 1 |
| HDGFRP3 | hepatoma-derived growth factor, related protein 3 |
| HLA-DMA | major histocompatibility complex, class II, DM alpha |
| HMBOX1 | homeobox containing 1 |
| HR | hairless homolog (mouse) |
| HSPB1 | heat shock 27kDa protein 1 |
| IGLL1 | immunoglobulin lambda-like polypeptide 1 |
| IL11RA | interleukin 11 receptor, alpha |
| ILF3 | interleukin enhancer binding factor 3, 90kDa |
| INMT | indolethylamine N-methyltransferase |
| IRAK1 | interleukin-1 receptor-associated kinase 1 |
| JUB | jub, ajuba homolog (Xenopus laevis) |
| KAZALD1 | Kazal-type serine peptidase inhibitor domain 1 |
| KCNQ1OT1 | KCNQ1 overlapping transcript 1 (non-protein coding) |
| KCTD11 | potassium channel tetramerisation domain containing 11 |
| KLHL12 | kelch-like 12 (Drosophila) |
| KRBA1 | KRAB-A domain containing 1 |
| LOC100129781 | hypothetical protein LOC100129781 |
| LOC100129846 | hypothetical protein LOC100129846 |
| LOC100130433 | hypothetical protein LOC100130433 |
| LOC100131857 | hypothetical protein LOC100131857 |
| LOC100132319 | hypothetical LOC100132319 |
| LOC100272228 | hypothetical LOC100272228 |
| LOC391334 | similar to Actin, cytoplasmic 1 |
| LOC442245 | glutathione S-transferase mu 2 pseudogene |
| LOC728392 | hypothetical protein LOC728392 |
| LOC729822 | similar to phosphatidylinositol transfer protein, cytoplasmic 1 |
| LOC731223 | hypothetical LOC731223 |
| LRRFIP2 | leucine rich repeat (in FLII) interacting protein 2 |
| MET | met proto-oncogene (hepatocyte growth factor receptor) |
| MICAL3 | microtubule associated monoxygenase, calponin and LIM domain containing 3 |
| MMD | monocyte to macrophage differentiation-associated |
| MPP4 | membrane protein, palmitoylated 4 (MAGUK p55 subfamily member 4) |
| MSH2 | mutS homolog 2, colon cancer, nonpolyposis type 1 (E. coli) |
| MT1X | metallothionein 1X |
| MYH9 | myosin, heavy chain 9, non-muscle |
| MYL9 | myosin, light chain 9, regulatory |
| MYO19 | myosin XIX |
| MYO19 | myosin XIX |
| NDFIP2 | Nedd4 family interacting protein 2 |
| NRIP3 | nuclear receptor interacting protein 3 |
| NUP43 | nucleoporin 43kDa |
| NUP85 | nucleoporin 85kDa |
| NYNRIN | NYN domain and retroviral integrase containing |
| PAQR6 | progestin and adipoQ receptor family member VI |
| PDGFRB | platelet-derived growth factor receptor, beta polypeptide |
| PEX11G | peroxisomal biogenesis factor 11 gamma |
| PF4 | platelet factor 4 |
| PHC2 | polyhomeotic homolog 2 (Drosophila) |
| PITPNC1 | phosphatidylinositol transfer protein, cytoplasmic 1 |
| PLCD1 | phospholipase C, delta 1 |
| PMAIP1 | phorbol-12-myristate-13-acetate-induced protein 1 |
| POFUT1 | protein O-fucosyltransferase 1 |
| POTEF | POTE ankyrin domain family, member F |
| PPTC7 | PTC7 protein phosphatase homolog (S. cerevisiae) |
| PSMD9 | proteasome (prosome, macropain) 26S subunit, non-ATPase, 9 |
| RAB2B | RAB2B, member RAS oncogene family |
| RARA | retinoic acid receptor, alpha |
| RASSF4 | Ras association (RalGDS/AF-6) domain family member 4 |
| RECK | reversion-inducing-cysteine-rich protein with kazal motifs |
| RPA2 | replication protein A2, 32kDa |
| RPA2 | replication protein A2, 32kDa |
| SDC1 | syndecan 1 |
| SELM | selenoprotein M |
| SEPP1 | selenoprotein P, plasma, 1 |
| SERPINH1 | serpin peptidase inhibitor, clade H (heat shock protein 47), member 1, (collagen binding protein 1) |
| SLC26A10 | solute carrier family 26, member 10 |
| SLC29A1 | solute carrier family 29 (nucleoside transporters), member 1 |
| SLC2A11 | solute carrier family 2 (facilitated glucose transporter), member 11 |
| SOCS6 | suppressor of cytokine signaling 6 |
| STARD10 | StAR-related lipid transfer (START) domain containing 10 |
| STAT2 | signal transducer and activator of transcription 2, 113kDa |
| STK39 | serine threonine kinase 39 (STE20/SPS1 homolog, yeast) |
| STRA13 | stimulated by retinoic acid 13 homolog (mouse) |
| SYNC | syncoilin, intermediate filament protein |
| SYT1 | synaptotagmin I |
| TAGAP | T-cell activation RhoGTPase activating protein |
| TAGLN | transgelin |
| TBC1D10B | TBC1 domain family, member 10B |
| TCEAL4 | transcription elongation factor A (SII)-like 4 |
| TFB1M | transcription factor B1, mitochondrial |
| TFPI | tissue factor pathway inhibitor (lipoprotein-associated coagulation inhibitor) |
| TFPI2 | tissue factor pathway inhibitor 2 |
| TGFBR2 | transforming growth factor, beta receptor II (70/80kDa) |
| TGM1 | transglutaminase 1 (K polypeptide epidermal type I, protein-glutamine-gamma-glutamyltransferase) |
| THBS2 | thrombospondin 2 |
| TIMM10 | translocase of inner mitochondrial membrane 10 homolog (yeast) |
| TMEM200A | transmembrane protein 200A |
| TTC21A | tetratricopeptide repeat domain 21A |
| TTLL3 | tubulin tyrosine ligase-like family, member 3 |
| TUBG1 | tubulin, gamma 1 |
| USP31 | ubiquitin specific peptidase 31 |
| VWA1 | von Willebrand factor A domain containing 1 |
| XRCC2 | X-ray repair complementing defective repair in Chinese hamster cells 2 |
| ZAK | sterile alpha motif and leucine zipper containing kinase AZK |
| ZFAND5 | zinc finger, AN1-type domain 5 |
| ZNF333 | zinc finger protein 333 |
| ZNF621 | zinc finger protein 621 |
| ZNF805 | zinc finger protein 805 |
| ***BPA-E2-TCDD common (n = 1)*** | |
| FBXW12 | F-box and WD repeat domain containing 12 |
