# Supplementary material for: Identification of Novel Low-Dose Bisphenol A Targets in Human Foreskin Fibroblast Cells Derived from Hypospadias Patients
Source: PLoS One. 2012 May 4;7(5):e36711. doi: 10.1371/journal.pone.0036711 (PMC3344929; doi:10.1371/journal.pone.0036711)
Supplement: Table S2 — KEGG pathways affected by BPA, E2 and TCDD identified by Pathway Express. (DOCX) [file pone.0036711.s006.docx]

| Chemical | KEGG pathways | P-Value |
| --- | --- | --- |
| BPA | ABC transporters | 0.120310768 |
|  | Basal cell carcinoma | 0.148086204 |
|  | Hedgehog signaling pathway | 0.153042577 |
|  | Long-term potentiation | 0.191683601 |
|  | Apoptosis | 0.228585739 |
|  | Melanogenesis | 0.257341716 |
|  | Leukocyte transendothelial migration | 0.29335649 |
|  | Natural killer cell mediated cytotoxicity | 0.325677539 |
|  | Wnt signaling pathway | 0.358422435 |
|  | Alzheimer''s disease | 0.405494492 |
|  | Cytokine-cytokine receptor interaction | 0.536857961 |
|  | Pathways in cancer | 0.619842831 |
| E2 | Colorectal cancer | 4.68701E-07 |
|  | p53 signaling pathway | 7.0906E-05 |
|  | Pathways in cancer | 7.93599E-05 |
|  | Prostate cancer | 0.000650041 |
|  | Chronic myeloid leukemia | 0.000683452 |
|  | Focal adhesion | 0.000962631 |
|  | Toll-like receptor signaling pathway | 0.001714048 |
|  | Basal cell carcinoma | 0.001882624 |
|  | Systemic lupus erythematosus | 0.002521461 |
|  | Adherens junction | 0.003660791 |
|  | Wnt signaling pathway | 0.003924219 |
|  | Regulation of actin cytoskeleton | 0.004802217 |
|  | B cell receptor signaling pathway | 0.004896941 |
|  | Cell cycle | 0.005263968 |
|  | ECM-receptor interaction | 0.005754239 |
|  | Endometrial cancer | 0.006412986 |
|  | MAPK signaling pathway | 0.007568837 |
|  | T cell receptor signaling pathway | 0.008343843 |
|  | Pancreatic cancer | 0.008544759 |
|  | VEGF signaling pathway | 0.009880498 |
|  | Small cell lung cancer | 0.021249332 |
|  | Hematopoietic cell lineage | 0.022488017 |
|  | Axon guidance | 0.024261599 |
|  | Apoptosis | 0.025113226 |
|  | Melanoma | 0.027081095 |
|  | Acute myeloid leukemia | 0.041555107 |
|  | Bladder cancer | 0.045919484 |
| TCDD | Homologous recombination | 1.71081E-07 |
|  | Nucleotide excision repair | 1.27261E-06 |
|  | Base excision repair | 1.84091E-06 |
|  | Cell cycle | 1.87174E-06 |
|  | Mismatch repair | 6.31541E-06 |
|  | Systemic lupus erythematosus | 0.000972157 |
|  | ECM-receptor interaction | 0.006433292 |
|  | p53 signaling pathway | 0.025949262 |
|  | Pathways in cancer | 0.047029486 |
