# Supplementary material for: Identification of Novel Low-Dose Bisphenol A Targets in Human Foreskin Fibroblast Cells Derived from Hypospadias Patients
Source: PLoS One. 2012 May 4;7(5):e36711. doi: 10.1371/journal.pone.0036711 (PMC3344929; doi:10.1371/journal.pone.0036711)
Supplement: Supplementary file 5 [file pone.0036711.s003.docx]

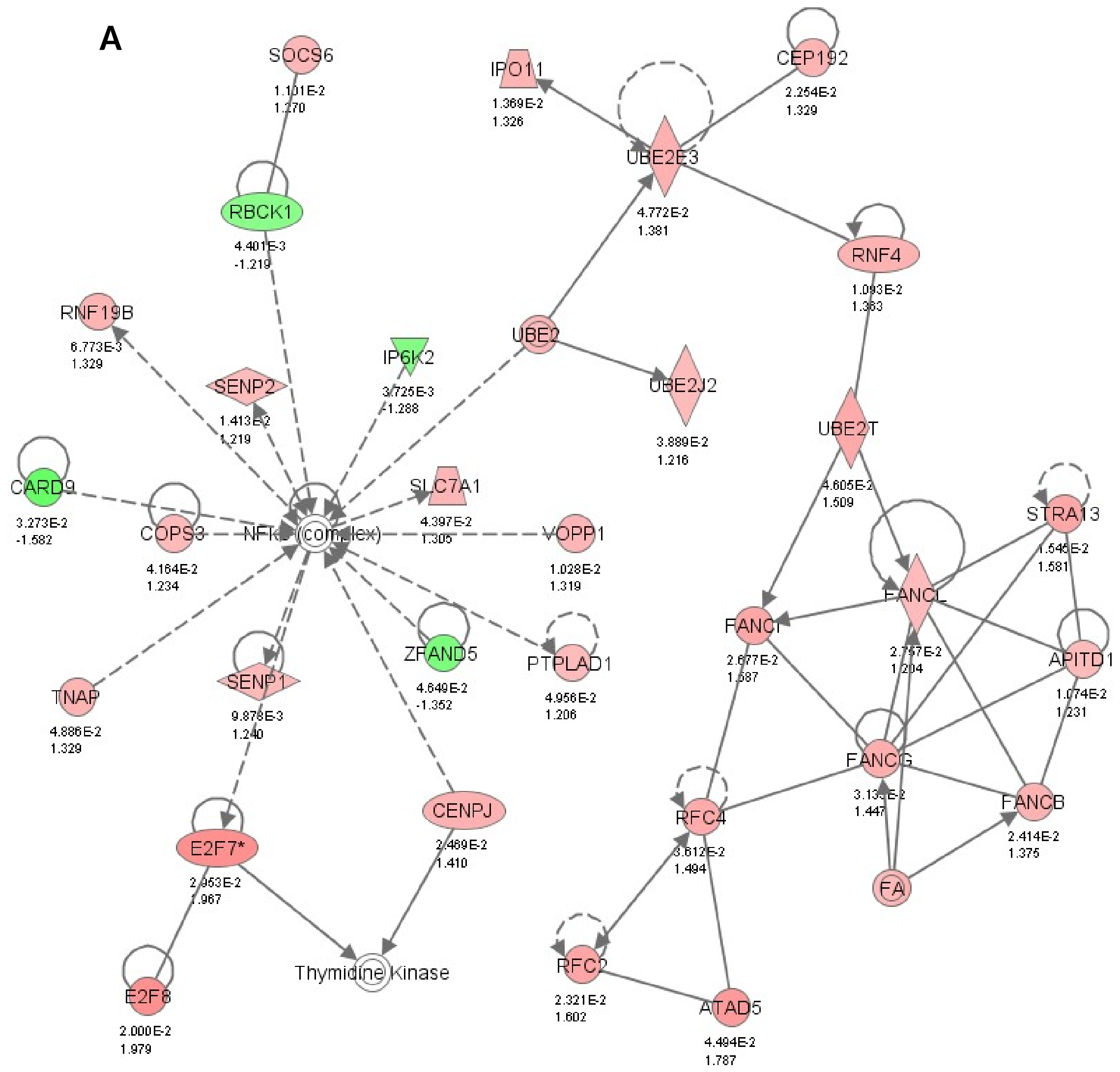

Fig S3. Qin *et al.*

B

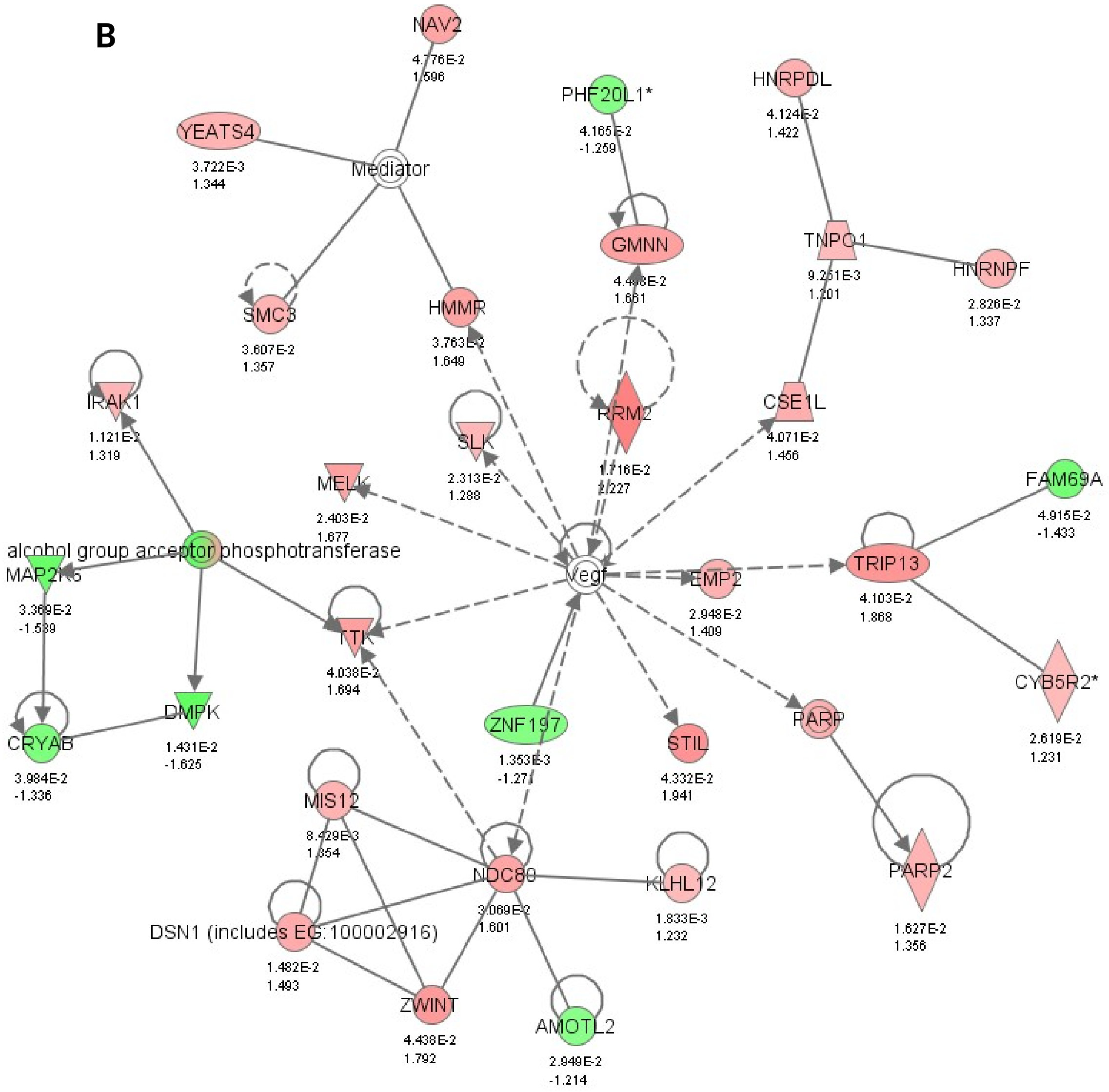

Fig S3. Qin *et al.*

C

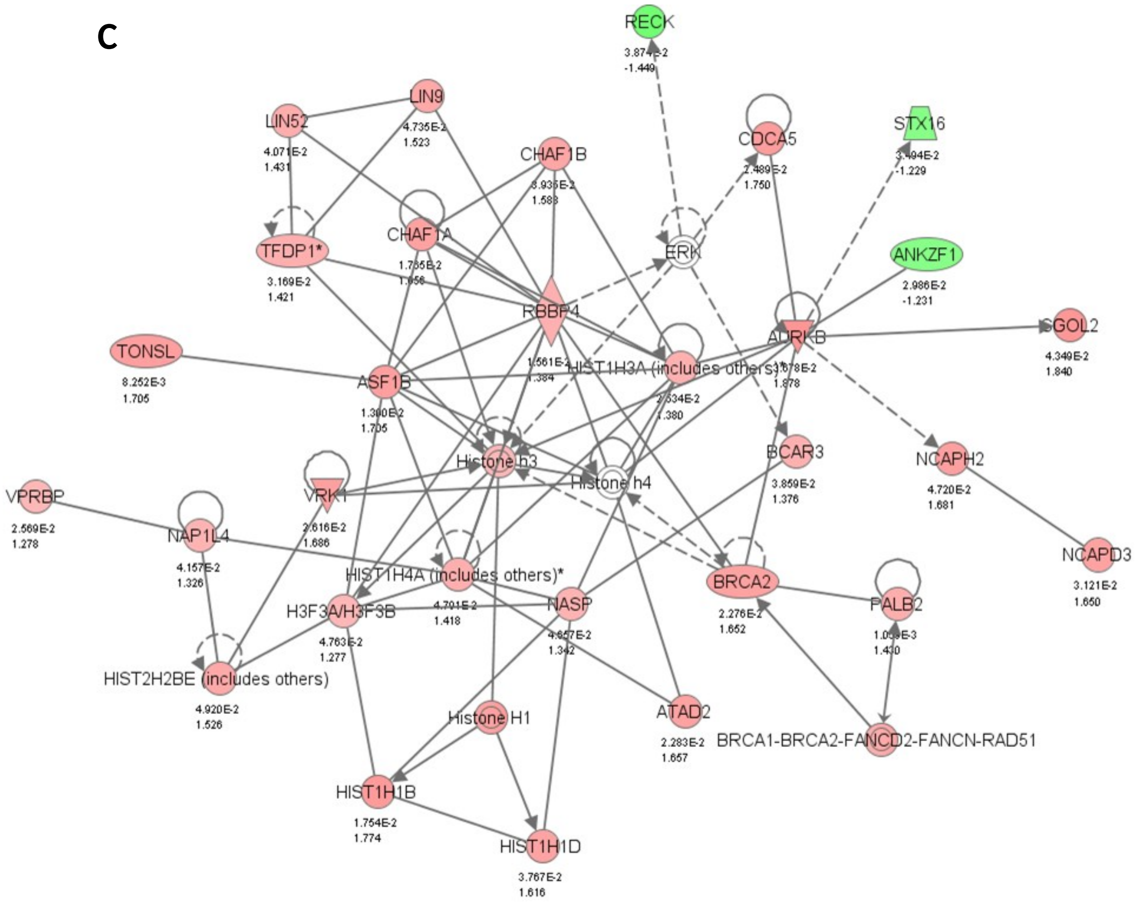

Fig S3. Qin *et al*.

D

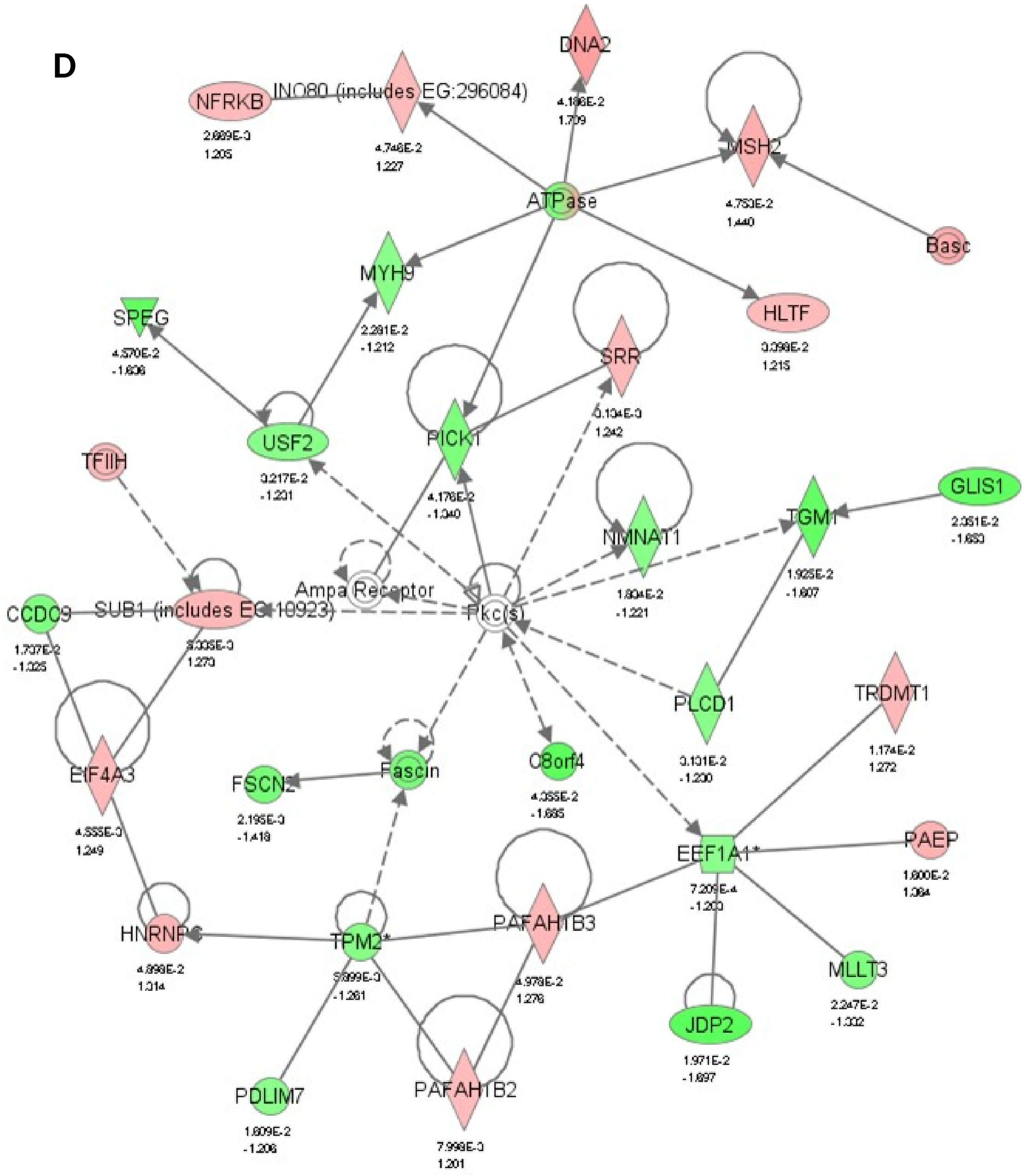

Fig S3. Qin *et al*.

E

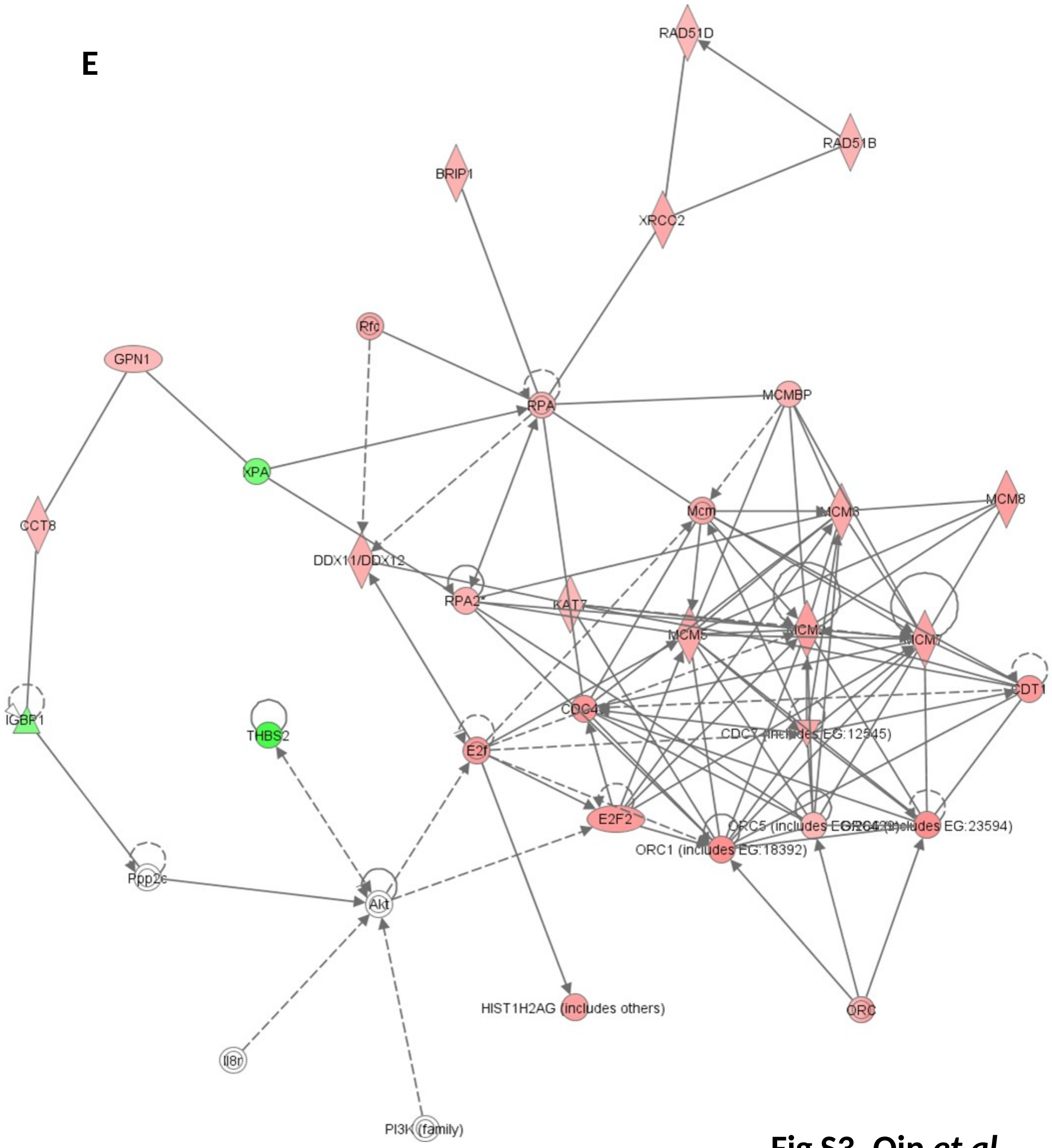

Fig S3. Qin *et al*.
